# Time‐lagged effects of habitat fragmentation on terrestrial mammals in Madagascar

**DOI:** 10.1111/cobi.13942

**Published:** 2022-09-20

**Authors:** Maarten J. E. Broekman, Jelle P. Hilbers, Aafke M. Schipper, Ana Benítez‐López, Luca Santini, Mark A. J. Huijbregts

**Affiliations:** ^1^ Department of Environmental Science, Radboud Institute for Biological and Environmental Sciences, Faculty of Science Radboud University Nijmegen The Netherlands; ^2^ PBL Netherlands Environmental Assessment Agency The Hague The Netherlands; ^3^ Integrative Ecology Group, Estación Biológica de Doñana (EBD‐CSIC) Sevilla Spain; ^4^ Department of Biology and Biotechnologies “Charles Darwin” Sapienza University of Rome Rome Italy; ^5^ National Research Council, Institute of Research on Terrestrial Ecosystems (CNR‐IRET) Rome Italy

**Keywords:** allometric relationships, biodiversity, extinction debt, fragmentation, habitat destruction, IUCN Red List, population persistence, biodiversidad, destrucción del hábitat, deuda de extinción, fragmentación, Lista Roja de la UICN, persistencia poblacional, relaciones alométricas, 异速关系, 生物多样性, 灭绝债务, 破碎化, 生境破坏, 《世界自然保护联盟红色名录》, 种群续存

## Abstract

Biodiversity is severely threatened by habitat destruction. As a consequence of habitat destruction, the remaining habitat becomes more fragmented. This results in time‐lagged population extirpations in remaining fragments when these are too small to support populations in the long term. If these time‐lagged effects are ignored, the long‐term impacts of habitat loss and fragmentation will be underestimated. We quantified the magnitude of time‐lagged effects of habitat fragmentation for 157 nonvolant terrestrial mammal species in Madagascar, one of the biodiversity hotspots with the highest rates of habitat loss and fragmentation. We refined species’ geographic ranges based on habitat preferences and elevation limits and then estimated which habitat fragments were too small to support a population for at least 100 years given stochastic population fluctuations. We also evaluated whether time‐lagged effects would change the threat status of species according to the International Union for the Conservation of Nature (IUCN) Red List assessment framework. We used allometric relationships to obtain the population parameters required to simulate the population dynamics of each species, and we quantified the consequences of uncertainty in these parameter estimates by repeating the analyses with a range of plausible parameter values. Based on the median outcomes, we found that for 34 species (22% of the 157 species) at least 10% of their current habitat contained unviable populations. Eight species (5%) had a higher threat status when accounting for time‐lagged effects. Based on 0.95‐quantile values, following a precautionary principle, for 108 species (69%) at least 10% of their habitat contained unviable populations, and 51 species (32%) had a higher threat status. Our results highlight the need to preserve continuous habitat and improve connectivity between habitat fragments. Moreover, our findings may help to identify species for which time‐lagged effects are most severe and which may thus benefit the most from conservation actions.

## Resumen

La biodiversidad se encuentra seriamente amenazada por la destrucción del hábitat. Como consecuencia de esta destrucción, el hábitat remanente se vuelve más fragmentado. Esto resulta en extirpaciones poblacionales retardadas dentro de los fragmentos restantes cuando éstos son muy pequeños para mantener a las poblaciones a largo plazo. Si se ignoran estos efectos retardados, se subestimarán los impactos a largo plazo de la pérdida del hábitat y la fragmentación. Cuantificamos la magnitud de los efectos retardados de la fragmentación del hábitat para 157 especies de mamíferos terrestres no voladores en Madagascar, uno de los puntos calientes de biodiversidad con las tasas más elevadas de pérdida del hábitat y fragmentación. Depuramos las extensiones geográficas de las especies con base en las preferencias de hábitat y los límites de elevación y después estimamos cuáles fragmentos de hábitat eran muy pequeños para mantener una población durante al menos cien años dadas las fluctuaciones estocásticas de las poblaciones. También analizamos si los efectos retardados cambiarían el estado de amenaza de la especie de acuerdo con el programa de evaluación de la Lista Roja de la UICN. Usamos relaciones alométricas para obtener los parámetros poblacionales requeridos para simular las dinámicas poblacionales de cada especie y cuantificamos las consecuencias de la incertidumbre en estos parámetros estimados mediante análisis repetidos con una gama de valores plausibles de los parámetros. Con base en los resultados promedio, descubrimos que para 34 especies (22% de las 157 especies) al menos el 10% de su hábitat actual tiene poblaciones inviables. Ocho especies (5%) cambiaron a un estado más elevado de amenaza cuando se consideraron los efectos retardados. Con base en los valores del centil 0.95, adherido a un principio precautorio, para 108 especies (32%) al menos el 10% de su hábitat tiene poblaciones inviables y 51 especies (32%) cambiaron negativamente su estado de amenaza. Nuestros resultados resaltan la necesidad de conservar la continuidad de los hábitats y mejorar la conectividad entre los fragmentos. Además, nuestros hallazgos pueden ayudar a identificar especies para las cuales los efectos retardados son más serios y que podrían beneficiarse más con las acciones de conservación.

## 【摘要】

生物多样性面临着栖息地破坏的严重威胁。栖息地破坏会导致剩余的栖息地破碎化更加严重, 进而在剩余破碎化栖息地的面积不足以长期支持种群发展时, 引发时间滞后的种群灭绝。如果忽视这种时滞效应, 那么将会低估栖息地丧失和破碎化的长期影响。马达加斯加是栖息地丧失和破碎化比例最高的生物多样性热点地区之一, 本研究量化了马达加斯加157种非飞行陆地哺乳动物栖息地破碎化的时滞效应。我们根据物种的栖息地偏好和海拔限制精细划分了其地理范围, 并估计在随机种群波动下, 哪些破碎化栖息地的面积不足以支持种群存活至少100年。我们还根据《世界自然保护联盟红色名录》的评估框架, 评估了时滞效应是否会改变物种的濒危等级。我们使用异速关系得到了每个物种种群动态模拟所需的种群参数, 并通过对一系列合理的参数值进行重复分析, 量化了这些参数估计的不确定性结果。我们发现基于中位数的结果, 有34个物种 (在157个物种中占22%) 当前的栖息地中至少有10% 包含难以存活的种群。如果考虑到时滞效应, 8个物种 (5%) 应被纳入更高的濒危等级。而基于0.95分位数的结果, 则有 108个物种 (69%) 在预防性原则下至少有10% 的栖息地包含难以存活的种群, 51个物种 (32%) 会被纳入更高的濒危等级。我们的结果强调了保护连续栖息地和提高破碎化栖息地连接度的必要性。此外, 我们的研究结果还有助于确定受时滞效应影响最严重且将从保护行动中受益最大的物种。**【翻译: 胡怡思; 审校: 聂永刚】**

## INTRODUCTION

Habitat destruction is currently one of the greatest threats to biodiversity (IPBES, 2019; Maxwell et al., 2016; Munstermann et al., 2021). Several recent studies have quantified the magnitude of habitat loss due to current and potential future habitat destruction, especially for mammals (e.g., Beyer & Manica, 2020; Gallego‐Zamorano et al., 2020; Powers & Jetz, 2019). However, habitat destruction results not only in habitat loss, but also in the fragmentation of the remaining habitat into small, isolated patches that host small, isolated populations (Didham, 2010; Ewers & Didham, 2006; Fahrig, 2003). When a population decreases below a certain threshold (i.e., minimum viable population size), it is unlikely to persist in the long term (Boyce, 1992; Shaffer, 1981). Some of the remaining habitat patches might thus be too small to support populations in the long run. Indeed, species responses to habitat conversion may not be immediate, and populations may persist for decades to centuries in habitat remnants before being extirpated (Figueiredo et al., 2019; Halley et al., 2016). If these time‐lagged effects are ignored, the ultimate effect of habitat loss on species persistence could be underestimated.

Time‐lagged extirpations are often referred to as extinction debt, which is defined as the number or proportion of species expected to become extinct in the future as the community reaches a new equilibrium after an environmental disturbance, such as habitat destruction and fragmentation (Kuussaari et al., 2009; Tilman et al., 1994). A recent literature review reports estimates of extinction debts ranging from 9% to 90% of current species richness (Figueiredo et al., 2019), highlighting the relevance of the phenomenon. However, most studies of extinction debt focus only on community‐level metrics, such as species richness (e.g., Chen & Peng, 2017; Cowlishaw, 1999; Semper‐Pascual et al., 2021; Wearn et al., 2012). To the best of our knowledge, no one has attempted to identify which species constitute the extinction debt. Identifying these species will provide a more complete picture of extinction debt and improve large‐scale conservation assessments. For example, one of the most commonly applied criteria in International Union for the Conservation of Nature (IUCN) Red List assessments, criterion B2, concerns area of occupancy (AOO) (i.e., area occupied by a species, excluding areas where vagrants occur) (Brooks et al., 2019; IUCN Standards and Petitions Committee, 2019). Because estimating occupancy at large geographic scales is rarely possible, typically the area of habitat (AOH) (i.e., the AOH within a species’ range [Brooks et al., 2019]) is taken as the upper bound of the AOO, under the assumption that the entire available habitat is occupied (Brooks et al., 2019; Santini et al., 2019; Tracewski et al., 2016). Yet, species are unlikely to fully occupy fragmented habitat because some fragments might be too small to host viable populations. Identifying these fragments may help refine assessments of species’ threat status. Knowledge of time‐lagged effects of habitat fragmentation may also help identify opportunities to take targeted conservation actions and prioritize conservation actions toward species that may benefit the most (Wearn et al., 2012).

We estimated the proportion of habitat area that is too small to host viable populations for 157 nonvolant terrestrial mammals of Madagascar. Madagascar is a biodiversity hotspot that has seen extensive habitat destruction over the last decades (Harper et al., 2007; Vieilledent et al., 2018) and hosts a large number of endemic species persisting in small fragments (Goodman & Benstead, 2005; Myers et al., 2000; Schwitzer et al., 2014). We therefore expected large time‐lagged effects of habitat fragmentation on Malagasy mammals. Further, we expected the time‐lagged effects to be particularly severe for forest specialist species because habitat destruction in Madagascar has resulted in large reductions and fragmentation of the original forest (Harper et al., 2007; Vieilledent et al., 2018).

## METHODS

### General approach and species selection

**FIGURE 1:**
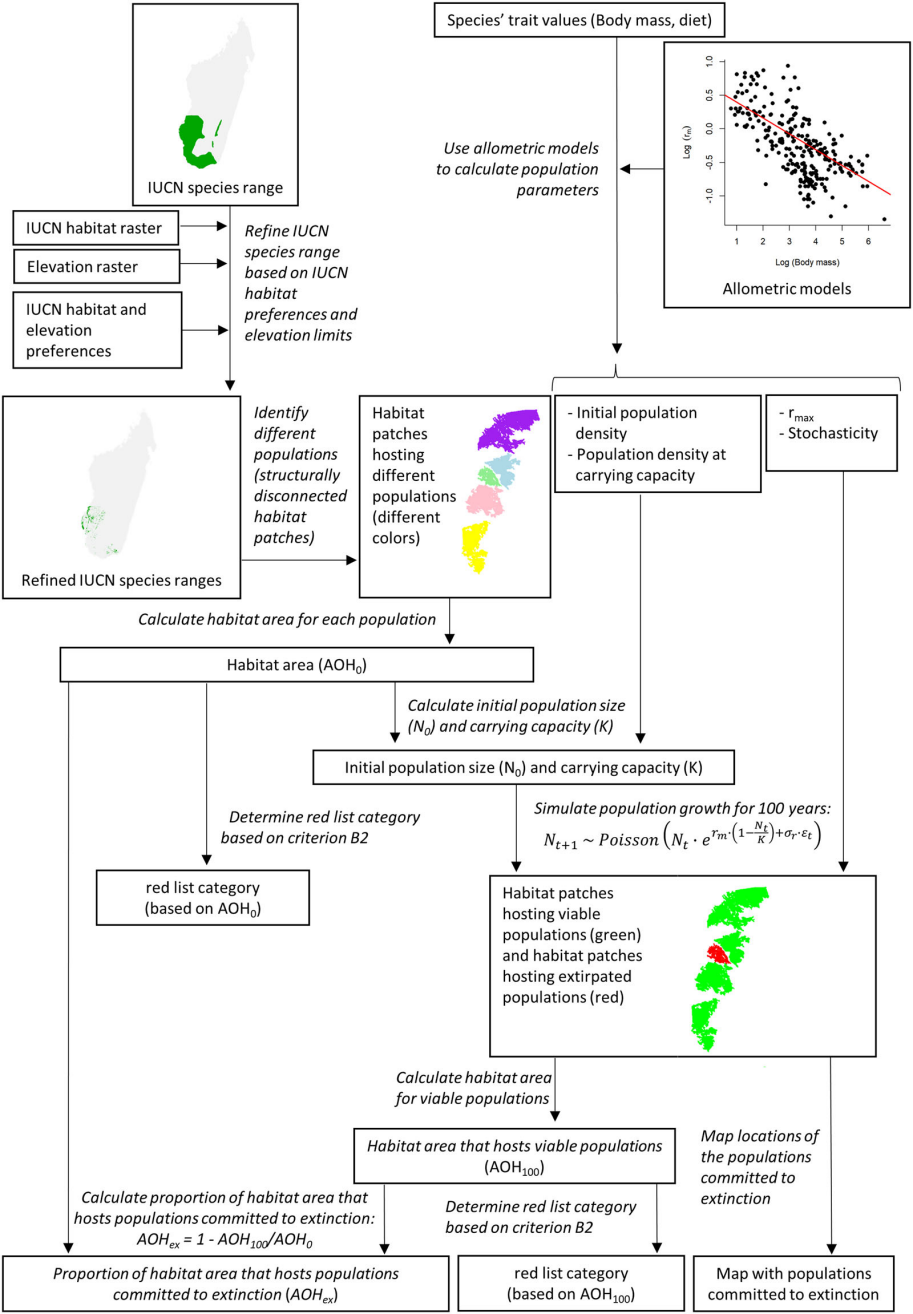
Overview of the analyses of time‐lagged effects of habitat fragmentation (white boxes, data; arrows, flow of data; italic text, short description of the analyses; IUCN, International Union for the Conservation of Nature). The IUCN and refined species range for the ring‐tailed lemur (*Lemur catta*) are shown. For illustrative purposes, only a subset of habitat patches are shown

We considered 157 species (96% of all endemic, nonvolant, terrestrial mammal species living in Madagascar), of which the majority were lemurs (93 species). We focused on nonvolant mammals because the allometric relationships included in our calculations would not be valid for bats (Duncan et al., 2007; Santini et al., 2013; Santini et al., 2018). We used habitat preferences published by IUCN (2021) to distinguish between forest specialist species and habitat generalist species. We defined forest specialists (124 species) as species occurring exclusively in forest habitat types and considered the remaining species (33) habitat generalists. For each species, we delineated AOH within its geographic range and then estimated which habitat fragments were too small to support a population for at least 100 years, given stochastic population fluctuations (Figure 1).

We used allometric relationships between required population parameters (i.e., initial population density, population density at carrying capacity, intrinsic population growth rate, environmental stochasticity, and median dispersal distance) and body mass to derive the input data required for the population simulations. The use of allometric relationships makes it possible to simulate population trajectories for a large number of species, which is otherwise not possible because the required population parameters are often unavailable and require detailed long‐term studies to estimate (Akçakaya & Sjögren‐Gulve, 2000; Beissinger & Westphal, 1998; Lacy, 2019).

Based on the population simulations, we quantified for each species the proportion of habitat that is too small to hold a viable population. We further used the results of the simulations to classify the species into IUCN Red List categories based on their AOO (criterion B2). As a third output, we produced a map of extinction debt based on the number of species committed to extirpation (Figure 1). Using this approach, we quantified time‐lagged effects in remaining habitat fragments specifically, ignoring potential extinction debt of species still occurring in areas that recently lost habitat.

### Area of habitat

To estimate the species’ AOH, we refined species’ geographical range as obtained from the IUCN (IUCN, 2021) based on their elevation limits and habitat preferences listed in the species’ assessments (e.g., Crooks et al., 2017; Gallego‐Zamorano et al., 2020; Santini et al., 2019). We used a global map of IUCN habitat types from 2015 at an ∼100‐m resolution (Jung et al., 2020) and the MERIT DEM elevation raster (Yamazaki et al., 2017) at 3 arc‐sec resolution (∼90 m). We resampled the latter to the resolution of the IUCN habitat types raster. We started from the assumption that existing habitat is entirely occupied; hence, our estimated AOH equaled the AOO (Brooks et al., 2019; Santini et al., 2019; Tracewski et al., 2016).

### Delineating populations

The AOH for a species often consists of several structurally disconnected habitat patches. Whether multiple patches host a single population depends on the movements of animals through the matrix. The majority of Malagasy mammals are forest specialists. For example, many lemurs are strictly arboreal, hence unlikely to cross, for example, nonforested areas. As a default, we therefore assumed that animals cannot disperse across structurally disconnected habitat patches (i.e., each habitat patch hosted a different population). However, because some species might be able to traverse the matrix to move between different habitat patches, we also considered a dispersal scenario. In this scenario, we assumed that patches within the median dispersal distance of a species host a single population, whereas patches farther away host different populations (Santini et al., 2019). To delineate populations in the dispersal scenario, we estimated the median dispersal distance of the species based on allometric relationships for carnivores and noncarnivores (Appendix S1).

### Population projections

We used the Ricker logistic growth equation to simulate the development of each population for 100 years, a commonly used time frame to estimate extinction probabilities in population viability analyses (Brook et al., 2006; Hilbers et al., 2017):

(1) $$N_{t+1}\;∼\;\mathrm{Poisson}\left(N_{t}\cdot e^{r_{m}\cdot \left(1-\frac{N_{t}}{K}\right)+\sigma_{r}\cdot \varepsilon_{t}}\right),$$

where *N_t_* and *N*_t+1_ are the population sizes in number of individuals at time *t* and *t* + 1, respectively, *K* is the population size at carrying capacity, *r_m_* is the intrinsic population growth rate per year, σ*_r_* is the standard deviation of *r_m_* (hereafter environmental stochasticity), and ε*_t_* is a term representing Gaussian white noise (mean = 0, variance = 1), which is randomly sampled at each time step. Because ε*_t_* is multiplied by the environmental stochasticity parameter, the random sampling of ε*_t_* simulates environmental stochasticity (Brook et al., 2006; Hilbers et al., 2017). We assumed that the sampled ε*_t_* value was similar for all populations of the same species because the species’ populations are generally close to each other and can therefore be assumed to be affected by environmental conditions in a similar way. To account for demographic stochasticity, we sampled the population size at time *t* + 1 from a Poisson distribution, taking the outcome of the Ricker logistic growth equation as lambda (Melbourne & Hastings, 2008). Following Hilbers et al. (2017), we assumed that populations could increase up to a maximum of 10% above their carrying capacity because higher values would result in highly negative values of the population growth rate and thus a collapse of the population.

We obtained *N*_0_ (initial population size) and *K* by multiplying the AOH for each population by the initial population density and the population density at carrying capacity, respectively. We assumed a constant population density for species across their AOH, thus ignoring potential edge effects. We consider this assumption justified because evidence suggests that Malagasy mammals (especially lemurs) are not edge sensitive (e.g., Lehman et al., 2006; Quemere et al., 2010; Wilmet et al., 2019). Because information on the population parameters (initial population density, population density at carrying capacity, intrinsic population growth rate, environmental stochasticity, and median dispersal distance) is lacking for many Malagasy mammals, we estimated these parameters based on allometric relationships (Appendix S1).

### Proportion of habitat area hosting populations committed to extirpation

For each species, we simulated all populations simultaneously and assumed that a population is lost when its size decreases below 2 individuals, the minimum number of individuals required to produce offspring in dioecious species. We then calculated the proportion of habitat area that hosts populations committed to extirpation as

(2) $${\;\mathrm{AOH}}_{\mathrm{ex}}=\;1-\frac{{\mathrm{AOH}}_{100}}{{\mathrm{AOH}}_{0}},$$

where AOH_ex_ is the proportion of habitat area that hosts a population committed to extirpation, AOH_0_ is the initial AOH, and AOH_100_ is the AOH that still hosts populations after 100 years.

To put our results on time‐lagged effects of habitat fragmentation into perspective, we compared them with projected future habitat losses in 2050 in 3 different habitat conversion scenarios corresponding to the shared socioeconomic pathways SSP1, SSP3, and SSP5 (O'Neill et al., 2017) (details on the quantification of future habitat loss in Appendix S2). This gives an indication of the magnitude of the time‐lagged effects of habitat fragmentation relative to expected future habitat losses.

### Red List classification

To evaluate the importance of time‐lagged effects of habitat fragmentation in terms of species’ threat status, we determined the red‐list category for each species according to criterion B2. To classify a species according to this criterion, the AOO of a species should be calculated as the area of 2 × 2 km grid cells in a species’ geographic range that contain habitat. A species is vulnerable if the AOO is <2000 km^2^, endangered if AOO is <500 km^2^, critically endangered if AOO is <10 km^2^, extinct if there is no occupied area, and least concern otherwise (IUCN Standards and Petitions Committee, 2019).

We assumed the AOO of species was equal to either AOH_0_ or AOH_100_ and compared the resulting red‐list categories. Following the IUCN guidelines, we resampled the habitat rasters at a 2 × 2 km resolution and assumed that a grid cell was occupied if it intersected any habitat (IUCN Standards and Petitions Committee, 2019). In reality, the AOO alone was not sufficient to assign a red‐list category because additional subcriteria had to be met (i.e., severely fragmented, continuing decline, extreme fluctuations). However, to illustrate the effect of considering time‐lagged effects in the red‐list assessments, we focused only on the AOO.

### Quantifying uncertainty

To quantify the uncertainty resulting from using the allometric models, we ran 10,000 simulations based on 100 different sets of population parameters (100 simulations for each set of population parameters). For the intrinsic population growth rate, environmental stochasticity, and the median dispersal distance, we randomly sampled 100 log_10_‐transformed values from a normal distribution with the value predicted by the allometric model as the mean and the root mean square error (RMSE) of the model as the standard deviation (Appendix S1). For the initial population densities and the population densities at carrying capacity, we randomly sampled 100 log_10_‐transformed values of both parameters simultaneously from a multivariate normal distribution because the residuals of the models predicting these densities were correlated (Spearman rank correlation coefficient of 0.70). We used the parameter values predicted with these models as the mean and the RMSE as the standard deviation. Furthermore, we used the Spearman rank correlation coefficients between the residuals of the density models as the covariance. We preferred the Spearman rank correlation coefficient over other correlation coefficients because it does not assume a linear relationship between variables and can therefore account for possible nonlinear relationships between the 2 sets of residuals.

To cover as much of the parameter space as possible while limiting the computational time, we first sampled 10^6^ random values for each of the population parameters as described above and then used a conditioned Latin hypercube sampling (Minasny & McBratney, 2006) to sample 100 combinations of population parameters from the original set of parameter combinations. To account for the correlation structure between the initial population density and the population density at carrying capacity, we based the conditioned Latin hypercube sampling on all population parameters, except the population density at carrying capacity.

To summarize the results of the 10,000 simulations for each species, we calculated for each species the median value of AOH_ex_ and determined the most frequent red‐list category. Following a precautionary principle, we also studied the 0.95 quantiles of AOH_ex_ and the highest red‐list category reached in at least 5% of the simulations.

To assess the contributions of the different model parameters to the uncertainty in the results, we calculated Spearman rank correlation coefficients between AOH_ex_ and each of the parameter values, separately for each species. We then scaled these correlation coefficients for each species (i.e., divided the squared correlation coefficient for each parameter by the sum of the squared correlation coefficients of all parameters, so their sum would equal 1). The larger the influence of a parameter on the result, the higher the correlation between the values of that parameter and the AOH_ex_ results. The resulting scaled correlation coefficients thus indicate the contribution of each parameter to the total variation in the proportion of habitat area that hosts population committed to extirpation due to time‐lagged effects. Finally, we averaged these relative contributions across all species.

### Hotspots of extinction debt

To identify potential spatial patterns in the magnitude of the time‐lagged effects of habitat fragmentation, we mapped for each species the populations with a probability of extirpation >5%. We then stacked the species maps to create a map of extinction debt (i.e., the number of species committed to extirpation). To account for spatial variation in species richness, we also generated a map with the extinction debt relative to the number of species initially present (based on their AOH). We decreased the resolution of these maps to 0.02 × 0.02 degrees by taking the maximum value of the smaller grid cells to improve visibility of areas with high absolute and relative numbers of populations committed to extirpation.

## RESULTS

### Proportion of habitat area hosting populations committed to extirpation

**FIGURE 2:**
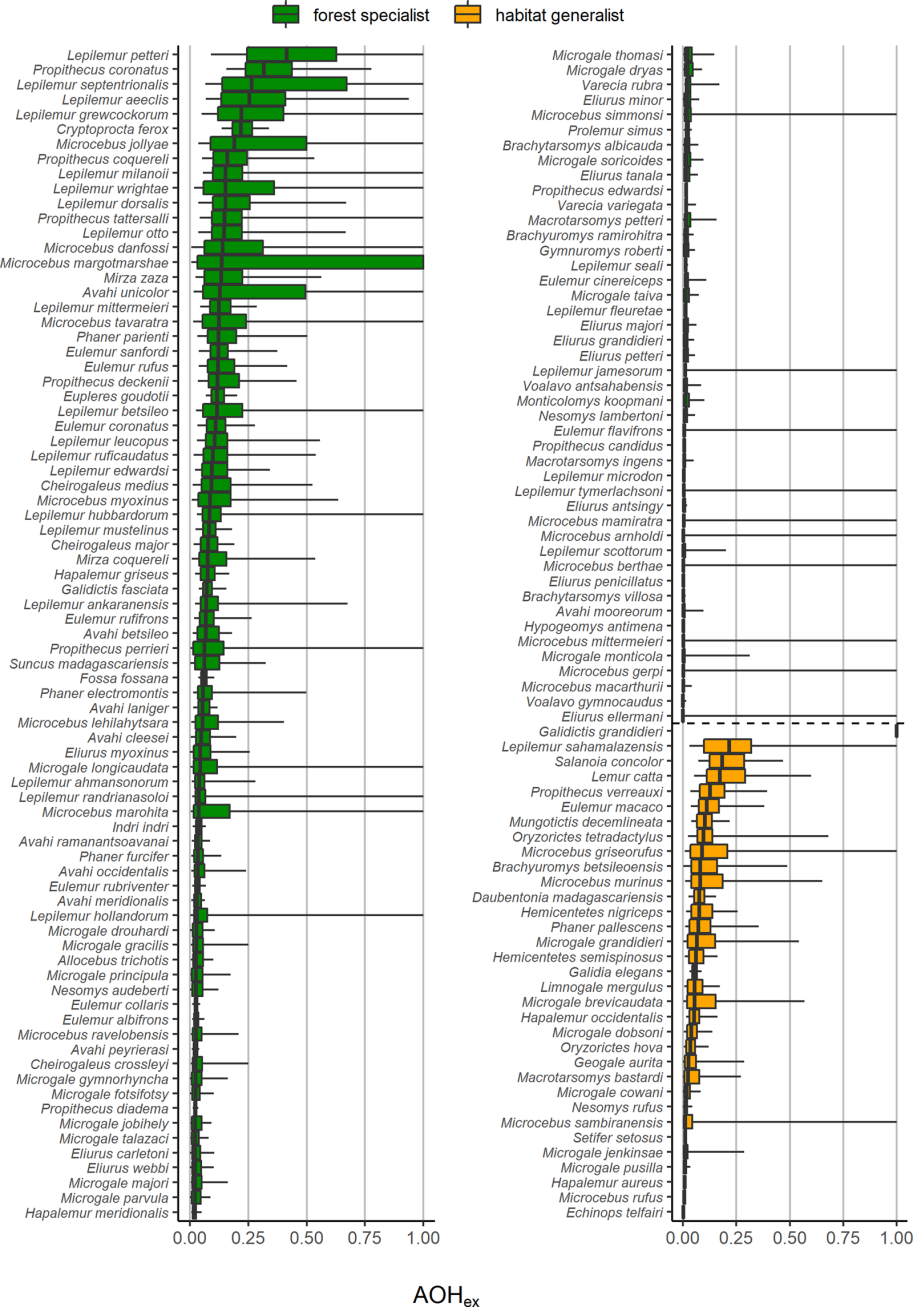
Proportion of habitat area that hosts populations committed to extirpation (AOH_ex_) based on 10,000 model simulations for forest specialist species and habitat generalists (boxes, 25th and 75th percentiles; vertical line in boxes, median; whiskers, 5th and 95th percentiles)

Based on median values for each species, we found that 5% of the 157 nonvolant mammal species were expected to lose at least 20% of their habitat area due to time‐lagged effects of habitat fragmentation (Figure 2; Appendices S3 & S4). In addition, 22% of the species were expected to lose at least 10% of their habitat area. For a considerable number of species, these time‐lagged effects of habitat fragmentation were larger than projected future habitat losses: 78%, 24%, and 29% of the species had larger time‐lagged effects of habitat fragmentation than projected future habitat loss in 2050 in scenarios SSP1, SSP3, and SSP5, respectively. For some species, time‐lagged effects of habitat fragmentation were more than 10 times larger than projected future habitat losses (Appendix S3).

**FIGURE 3:**
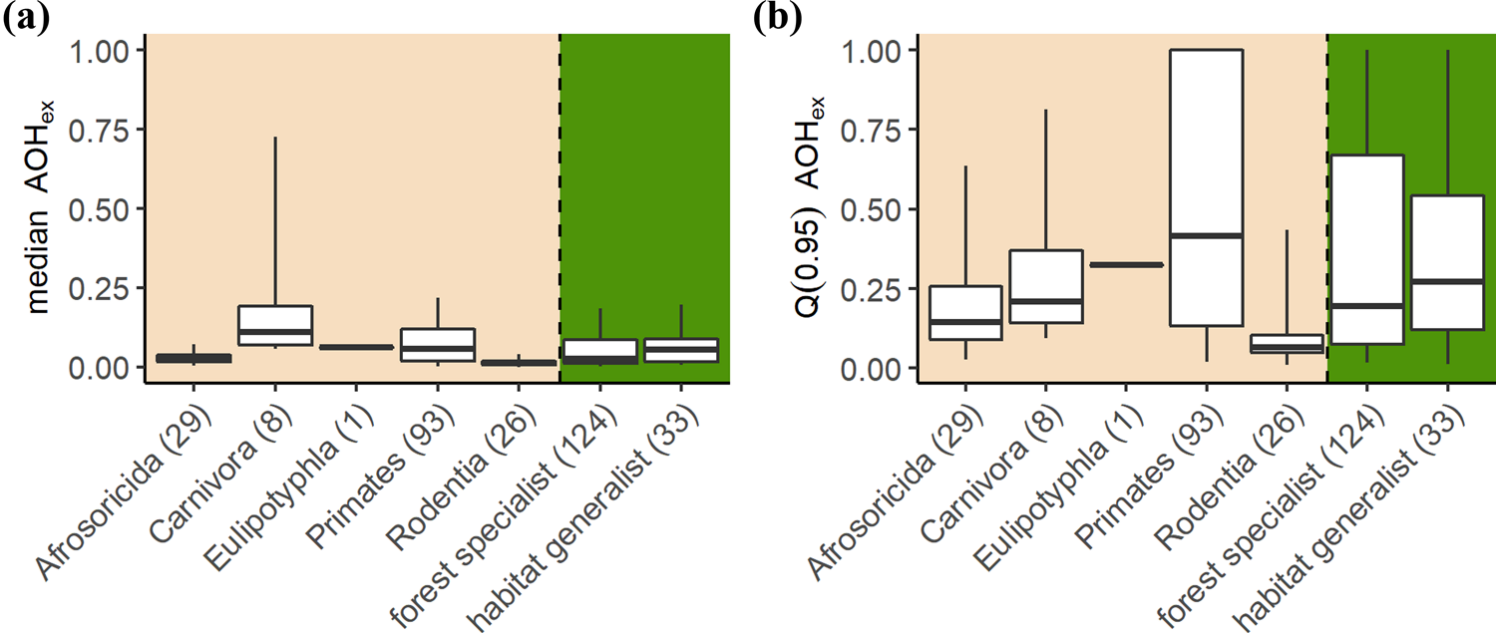
Variation in the (a) median and (b) 0.95‐quantile value of the proportion of habitat area that hosts populations committed to extirpation (AOH_ex_) for species from different taxonomic orders and for forest specialist and habitat generalist species (boxes, 25th and 75th percentiles; vertical line in boxes, median; whiskers, 5th and 95th percentiles)

The time‐lagged effects of habitat fragmentation were comparable for forest specialists (22% of the species expected to lose at least 10% of their habitat area) and habitat generalists (21% of the species expected to lose at least 10% of their habitat area), but differed among taxonomic orders (Figure 3a). The largest proportions of habitat area hosting populations committed to extirpation were for species from the orders Carnivora and Primates. For example, for the grandidier's vontsira (*Galidictis grandidieri*), we predicted that the time‐lagged effects of habitat fragmentation would lead to the extinction of the species because all of its remaining habitat patches were expected to be too small to sustain a viable population. For the petter's sportive lemur (*Lepilemur petteri*), 42% of its habitat area hosted populations committed to extirpation. When we assumed that species can disperse through the matrix, the proportions of habitat area hosting populations committed to extirpation were much lower, and only 2% of the species were expected to lose at least 10% of their habitat area (Appendices S3 & S4). However, even in the dispersal scenario for some species the time‐lagged effects of habitat fragmentation were considerable (e.g., a median value of 100% for grandidier's vontsira and 19% for petter's sportive lemur). Also in the dispersal scenario, there was no difference between forest specialists and habitat generalists, and the largest proportions of habitat area hosting populations committed to extirpation were still found for species from the orders Carnivora and Primates (Appendix S4).

Following a precautionary principle, by studying the 0.95 quantiles of AOH_ex_ (i.e., the upper whiskers of Figure 2), we found much larger time‐lagged effects of habitat fragmentation: 69% of species were expected to lose at least 10% of their habitat area. In addition, 32 species were likely to become globally extinct based on a precautionary interpretation of the predicted AOH_ex_. The spread in the AOH_ex_ within each species was mostly attributed to uncertainty in the environmental stochasticity parameter (on average 39% across all species) and the density at carrying capacity (37%), followed by uncertainty in the initial population density (20%) and intrinsic population growth rate (4%).

### Red‐list classification

**FIGURE 4:**
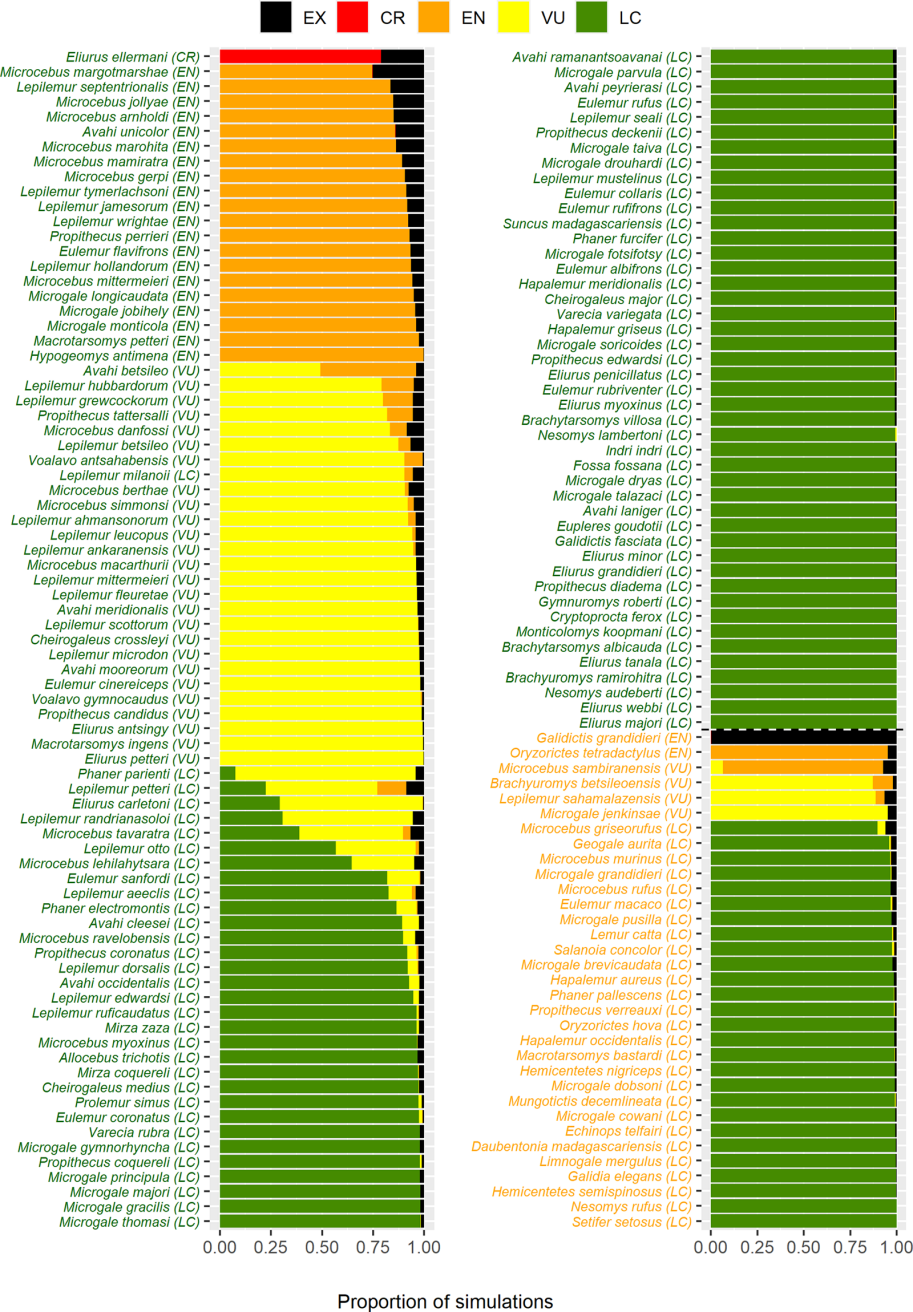
Proportion of the 10,000 simulations in which a species is assigned a different International Union for the Conservation of Nature (IUCN) Red List category based on IUCN criterion B2, assuming the area of occupancy is equal to the habitat area that hosts viable populations (AOH_100_). Categories in parentheses indicate a species’ category based on IUCN criterion B2, assuming the area of occupancy is equal to the total habitat area (AOH_0_) (green type, forest specialist species; orange type, habitat generalist species; LC, least concern; NT, near threatened; VU, vulnerable; EN, endangered; CR, critically endangered; EX, extinct)

Eight species (5%), including 6 forest specialist species, were categorized in a higher red‐list category when we used their habitat area hosting viable populations as the AOO, instead of the total initial habitat area (Figure 4). In the dispersal scenario, this was the case for 4 species (3%; Appendices S3 & S4), including 2 forest specialist species.

When we followed a precautionary principle and used the highest red‐list category that could be reached in at least 5% of the simulations, the numbers of species with higher red‐list categories changed to 51 (32%).

### Hotspots of extinction debt

**FIGURE 5:**
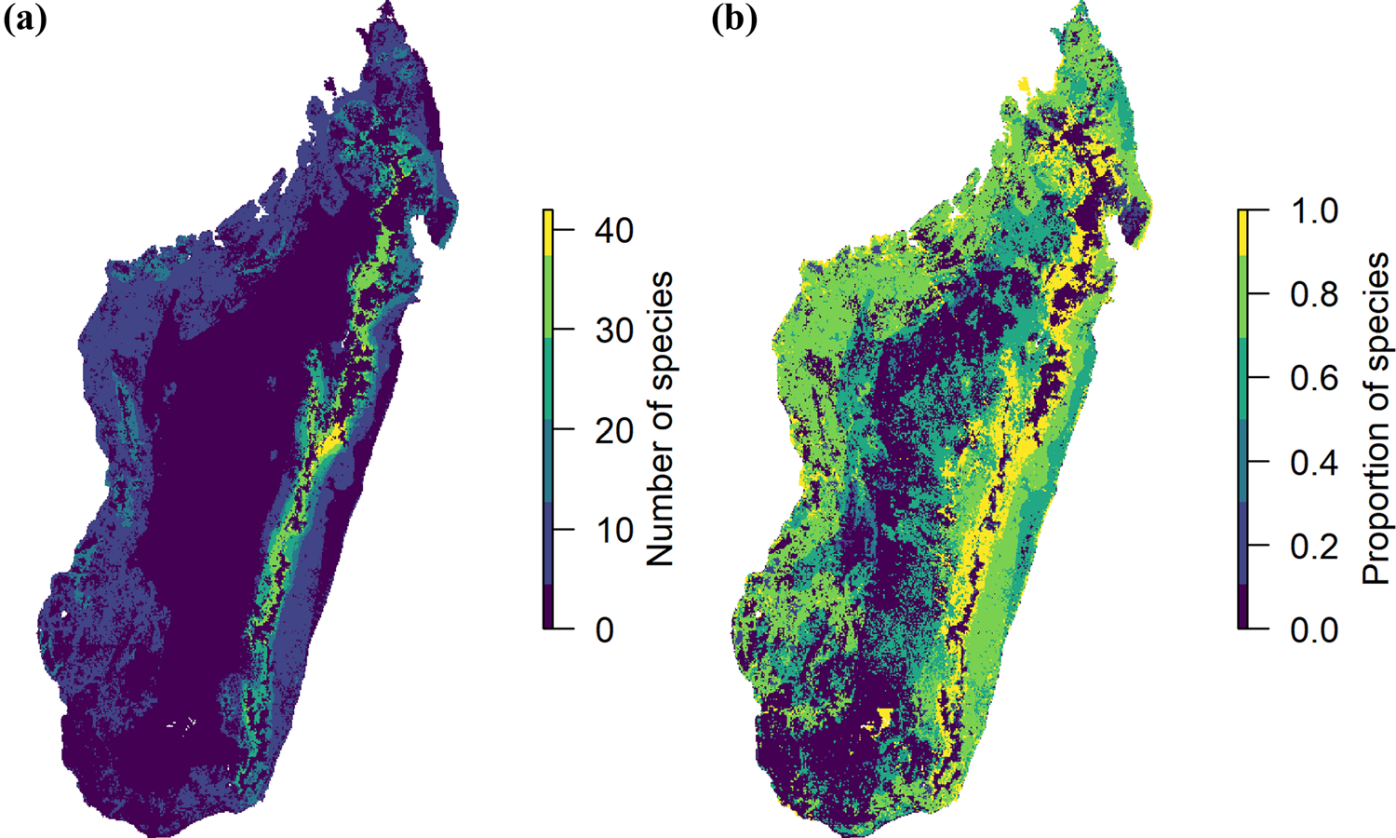
In Madagascar (a) absolute and (b) relative number of species committed to extirpation (probability of extirpation within 100 years >5%)

The largest time‐lagged effects were in eastern Madagascar, especially around the edges of the remnant rainforest and littoral forest (Figure 5). Areas with >50% of the species having populations committed to extirpation occurred almost everywhere in Madagascar, except the southern and central regions. Spatial patterns of extinction debt were similar for the dispersal scenario (Appendix S4).

## DISCUSSION

For 34 of the 157 (22%) Malagasy mammals in our study, we found considerable time‐lagged effects of habitat fragmentation (>10% of current AOH contains unviable populations), possibly much larger than projected future habitat losses. The areas with the largest time‐lagged effects of habitat fragmentation were mostly in eastern Madagascar around the edges of the remnant rainforest and littoral forest and largely corresponded to areas where deforestation is taking place (Vieilledent et al., 2018). These results are concerning because Madagascar's biodiversity is already severely threatened by habitat loss without considering time‐lagged effects of habitat fragmentation (e.g., Habel et al., 2019; Morelli et al., 2020; Schwitzer et al., 2014; Vieilledent et al., 2018). For example, the critically endangered northern sportive lemur (*Lepilemur septentrionalis*) and crowned sifaka (*Propithecus coronatus*) have lost over 80% of their individuals to habitat loss during their last 3 generations (IUCN, 2021). We estimated that these species may lose an additional 27% and 32%, respectively, of their remaining population due to time‐lagged effects.

We further found that time‐lagged effects of habitat fragmentation are not limited to forest specialist species. For example, the habitat of grandidier's vontsira, which is expected to lose all its habitat due to time‐lagged effects, consists of shrubland. However, for all habitat generalist Malagasy mammals included in the analyses, forest is also part of their habitat (IUCN, 2021). In general, the largest time‐lagged effects of habitat fragmentation were for Primates and Carnivora. These orders contain the largest mammal species in Madagascar, which have the lowest population densities (Santini et al., 2018). The resulting low population sizes of these species make them prone to extirpation, which might explain why species from these orders have the largest time‐lagged effects due to habitat fragmentation. A previous study also found that large‐bodied species were more vulnerable to extinction (Hilbers et al., 2016).

The evaluation of red‐list categories according to criterion B2 underpins the importance of understanding time‐lagged effects of habitat fragmentation. For example, the Ankarana special reserve tufted‐tailed rat (*Eliurus carletoni*) is classified as least concern based on all habitat area, but when we considered only the habitat area that hosts viable populations, it was classified as vulnerable in 70% of the simulations. Our findings imply a potentially high payoff of conservation actions for this particular species because such actions could prevent the time‐lagged effects of habitat fragmentation. This example highlights the importance of considering which habitat fragments are able to support viable populations in IUCN Red List assessments and of identifying related conservation actions.

Our results were associated with relatively large uncertainty. For example, the number of species for which >10% of the habitat will be lost due to time‐lagged effects increased from 34 to 108 when we use the 0.95 quantiles of AOH_ex_ for each species, instead of the median value. This uncertainty reflects the uncertainty in the allometric estimates of the population parameters combined with the inherent stochasticity of the Ricker logistic model, which includes Gaussian white noise. This uncertainty is the price of generality. Nevertheless, despite this uncertainty, our results still allowed us to make some important conclusions. For example, the results based on the median AOH_ex_ values showed that almost one‐quarter of the species will lose an additional 10% of habitat due to time‐lagged effects. In addition, when time‐lagged effects were considered, 8 species were assigned higher red‐list categories.

To estimate the sensitivity of the results to the use of allometric relationships to estimate population parameters, instead of using empirical estimates, we also ran our models with empirical, species‐specific values of the population parameters for 24 species (Appendix S5). For these species, an empirical value was available for at least 1 of the population parameters. Running the models based on these species‐specific parameters hardly affected our results (Appendix S5), reflecting that the empirical data were typically well within the 90% confidence interval of the allometric estimates (Appendix S5). Nevertheless, we could not find empirical estimates for the most uncertain parameter (i.e., environmental stochasticity).

Our results complement the findings from other empirical and theoretical studies on extinction debts that show time‐lagged effects of habitat destruction and fragmentation can be large and may be even larger than its immediate effects (e.g., Halley et al., 2014; Wearn et al., 2012). Compared with methods used previously in studies of time‐lagged effects, our method offers the advantage of a focus on individual species, instead of community patterns. We were therefore able to identify species expected to be most affected by time‐lagged effects of habitat fragmentation, such as the grandidier's vontsira, petter's sportive lemur, and the Ankarana special reserve tufted‐tailed rat, in Madagascar. This information may inform prioritization of species for more refined extinction risk assessments and conservations actions.

The large time‐lagged effects of habitat fragmentation we found imply that conservation actions should not just involve conserving existing habitat. Even without any further direct habitat losses, populations are expected to be lost due to time‐lagged effects. Therefore, we recommend restoration of fragmented habitat and improvement of connectivity between habitat fragments with wildlife corridors (e.g., implementation of forest regeneration enhancement plans around small isolated patches). This may increase the habitat area for species and thus their probability of survival. Secondary forests may not be as valuable for biodiversity as primary forests (Gibson et al., 2011), but lemurs have been observed at high densities in regenerating secondary forests (Miller et al., 2018) and are able to adapt to new environments (Donati et al., 2020). Targeted conservation actions that account for potential time‐lagged effects of habitat fragmentation could thus contribute to the conservation of Madagascar's unique biodiversity.

## Supporting information

Additional supporting information may be found in the online version of the article at the publisher's website.

Additional supporting information may be found in the online version of the article at the publisher's website.

Additional supporting information may be found in the online version of the article at the publisher's website.

Additional supporting information may be found in the online version of the article at the publisher's website.

Additional supporting information may be found in the online version of the article at the publisher's website.
