# Supplementary material for: Time‐lagged effects of habitat fragmentation on terrestrial mammals in Madagascar
Source: Conserv Biol. 2022 Sep 20;36(5):e13942. doi: 10.1111/cobi.13942 (PMC9826438; doi:10.1111/cobi.13942)
Supplement: Supplementary file 1 — Additional supporting information may be found in the online version of the article at the publisher's website. [file COBI-36-0-s003.docx]

APPENDIX S1. ALLOMETRIC MODELS

We estimated a species’ intrinsic population growth rate (r_m_), standard deviation of the intrinsic population growth rate (environmental stochasticity, σ_r_), median dispersal distance (Disp), initial population density (D_initial_) and population density at carrying capacity (D_K_) using allometric relationships between these log_10_-transformed parameters and the log_10_-transformed species’ body mass (Table S1):

log_10_(r_m_) ~ log_10_(BM) + (1|Order/Family)

log_10_(σ_r_) ~ log_10_(BM) + (1|Order/Family)

log_10_(Disp_carnivores_) ~ log_10_(BM) + (1|Order/Family)

log_10_(Disp_non-carnivores_) ~ log_10_(BM) + (1|Order/Family)

log_10_(D_initial_) ~ log_10_(BM) + (1|Order/Family)

log_10_(D_K_) ~ log_10_(BM) + (1|Order/Family)

To account for taxonomic relatedness we included family and order as random effects in these models. The root mean square error (RMSE) of these models was calculated by taking the square root of the average squared difference between the observed parameter values in the datasets used to fit the models and the parameter values predicted with the fitted models.

Table S1. Coefficients and the root mean square error (RMSE) of the linear mixed-effects models for all population parameters, as well as the number of observations used to fit the model (n).

| Parameter | *n* | Intercept (SE) | Slope (SE) | RMSE |
| --- | --- | --- | --- | --- |
| Initial population density | 255 | 2.59 (0.33) | -0.46 (0.07) | 0.52 |
| Population density at carrying capacity | 255 | 3.16 (0.34) | -0.41 (0.07) | 0.53 |
| Intrinsic population growth rate (r_m_) | 251 | 0.63 (0.10) | -0.24 (0.02) | 0.15 |
| Environmental stochasticity ($\sigma_{r}$) | 93 | 0.31 (0.07) | -0.19 (0.02) | 0.22 |
| Median dispersal distance (carnivores) | 21 | -0.49 (0.47) | 0.43 (0.12) | 0.42 |
| Median dispersal distance (non-carnivores) | 49 | -1.43 (0.38) | 0.48 (0.10) | 0.46 |

To fit models for the intrinsic population growth rate and the environmental stochasticity we used data from Duncan et al. (2007) and Brook et al. (2006). Duncan et al. (2007) report species’ intrinsic population growth rates, estimated using two different methods: (1) estimating r_m_ from log-transformed temporal changes in estimates of abundance (ln(N_t+1_/N_t_)) when resources are not limiting (Caughley, 1977), or (2) estimating r_m_  by solving Cole’s equation (Cole, 1954) using three life-history parameters: age at first reproduction, annual fecundity, and age at last reproduction. The estimates of intrinsic population growth rate, are thus estimated from field data and not based on extrapolations

To fit a model for median dispersal distance we used data from Whitmee & Orme (2013) and Santini et al. (2013) and fitted a separate model for carnivores and non-carnivores, as both these studies revealed that body mass affected median dispersal distance differently in carnivores and non-carnivores. Some species had multiple estimates of intrinsic population growth rate and median dispersal distance in these datasets, in which case we took the median to retain only one estimate per species.

To fit models for the initial population density and population density at carrying capacity, we used data from TetraDENSITY. Because population densities can vary both spatially and temporally, we only retained species with at least 10 density estimates. For species with a lower number of density estimates, it is difficult to establish whether the population densities represent a maximum density value (population density at carrying capacity) or a lower population density.

For the initial population density we then fitted a model between the log_10_ of the median density and the log_10_ of the body mass and for the population density at carrying capacity we fitted a model between the log_10_ of the 0.95 quantile of the densities and the log_10_ of the body mass. As diet (i.e., whether the species is classified as a carnivore, herbivore or omnivore), and quadratic and cubic effects of body mass might also explain variation in species density, we fitted models including these effects as well and performed model selection based on the Bayesian information criterion (BIC) to select the most parsimonious model (Santini et al., 2018b). The most parsimonious model contained only an effect of the body mass for both the initial population density and the density at carrying capacity (Tables S2-S3).

Table S2. Fitted models to predict the log_10_ of the initial population density with their BIC values and RMSE’s.

| Model* | BIC | RMSE |
| --- | --- | --- |
| log_10_(D_initial_) ~ log_10_(BM) + (1\|Order/Family) | 530.0 | 0.52 |
| log_10_(D_initial_) ~ log_10_(BM) + log_10_(BM)^2^ + (1\|Order/Family) | 538.4 | 0.52 |
| log_10_(D_initial_) ~ log_10_(BM) + Diet + (1\|Order/Family) | 538.5 | 0.51 |
| log_10_(D_initial_) ~ log_10_(BM) + log_10_(BM)^2^ + log_10_(BM)^3^ + (1\|Order/Family) | 546.7 | 0.51 |
| log_10_(D_initial_) ~ log_10_(BM) + log_10_(BM)^2^ + Diet + (1\|Order/Family) | 547.1 | 0.51 |
| log_10_(D_initial_) ~ Diet + (1\|Order/Family) | 556.5 | 0.50 |
| log_10_(D_initial_) ~ log_10_(BM) + log_10_(BM)^2^ + log_10_(BM)^3^ + Diet + (1\|Order/Family) | 556.5 | 0.51 |

*D_initial_ indicates the initial population density in individuals/km^2^, BM indicates the body mass in grams, and Diet indicates whether the species is a carnivore, omnivore or herbivore.

Table S3. Fitted models to predict the log_10_ of the density at the carrying capacity with their BIC values and RMSE’s.

| Model* | BIC | RMSE |
| --- | --- | --- |
| log_10_(D_K_) ~ log_10_(BM) + (1\|Order/Family) | 536.1 | 0.53 |
| log_10_(D_K_) ~ log_10_(BM) + Diet + (1\|Order/Family) | 542.3 | 0.52 |
| log_10_(D_K_) ~ log_10_(BM) + log_10_(BM)^2^ + (1\|Order/Family) | 542.7 | 0.52 |
| log_10_(D_K_) ~ log_10_(BM) + log_10_(BM)^2^ + Diet + (1\|Order/Family) | 549.1 | 0.52 |
| log_10_(D_K_) ~ log_10_(BM) + log_10_(BM)^2^ + log_10_(BM)^3^ + (1\|Order/Family) | 551.3 | 0.52 |
| log_10_(D_K_) ~ Diet + (1\|Order/Family) | 557.0 | 0.51 |
| log_10_(D_K_) ~ log_10_(BM) + log_10_(BM)^2^ + log_10_(BM)^3^ + Diet + (1\|Order/Family) | 558.6 | 0.52 |

*D indicates the population density at the carrying capacity in individuals/km^2^, BM indicates the body mass in grams, and Diet indicates whether the species is a carnivore, omnivore or herbivore.

Body mass and diet data for Malagasy mammals, used to predict population parameters with these allometric relationships, were obtained from MADA (Razafindratsima et al., 2018) which is the most recent and complete dataset of functional trait data of Malagasy mammals. For species with missing data in MADA, we resorted to other datasets (Wilman et al., 2014; Santini et al., 2018a). For the Ankarana special reserve tufted-tailed rat (*Eliurus carletoni*) no diet data were available in any of these datasets, so for this species we used the most frequent diet from congeneric species (Appendix S4).

LITERATURE CITED

Brook B.W., Traill L.W., Bradshaw C.J.A. (2006) Minimum viable population sizes and global extinction risk are unrelated. *Ecology Letters*, 9, 375-382.

Caughley G. (1977) Analysis of vertebrate populations. John Wiley, New York, USA.

Cole L.C. (1954) The population consequences of life history phenomena. *The Quarterly review of biology*, 29, 103-137.

Duncan R.P., Forsyth D.M., Hone J. (2007) Testing the metabolic theory of ecology: Allometric scaling exponents in mammals. *Ecology*, 88, 324-333.

Razafindratsima O.H., Yacoby Y., Park D.S. (2018) MADA: Malagasy Animal trait Data Archive. *Ecology*, 99, 990-990.

Santini L., di Marco M., Visconti P., Baisero D., Boitani L., Rondinini C. (2013) Ecological correlates of dispersal distance in terrestrial mammals. *Hystrix-Italian Journal of Mammalogy*, 24, 181-186.

Santini L., Isaac N.J.B., Ficetola G.F. (2018a) TetraDENSITY: A database of population density estimates in terrestrial vertebrates. *Global Ecology and Biogeography*, 27, 787-791.

Santini L., Isaac N.J.B., Maiorano L., Ficetola G.F., Huijbregts M.A.J., Carbone C., Thuiller W. (2018b) Global drivers of population density in terrestrial vertebrates. *Global Ecology and Biogeography*, 27, 968-979.

Whitmee S., Orme C.D.L. (2013) Predicting dispersal distance in mammals: a trait-based approach. *Journal of Animal Ecology*, 82, 211-221.

Wilman H., Belmaker J., Simpson J., de la Rosa C., Rivadeneira M.M., Jetz W. (2014) EltonTraits 1.0: Species‐level foraging attributes of the world's birds and mammals: Ecological Archives E095‐178. *Ecology*, 95, 2027-2027.
