# Supplementary material for: Time‐lagged effects of habitat fragmentation on terrestrial mammals in Madagascar
Source: Conserv Biol. 2022 Sep 20;36(5):e13942. doi: 10.1111/cobi.13942 (PMC9826438; doi:10.1111/cobi.13942)
Supplement: Supplementary file 2 — Additional supporting information may be found in the online version of the article at the publisher's website. [file COBI-36-0-s005.docx]

APPENDIX S2. PROJECTED FUTURE HABITAT LOSS

We estimated projected future habitat losses for Malagasy mammals in 2050 in three different habitat conversion scenarios, corresponding to the Shared Socioeconomic Pathways SSP1, SSP3 and SSP5 (O'Neill et al., 2017).

**Shared socioeconomic pathways**

Scenario SSP1 represents the “sustainability” scenario in which the growth of the human population and the use of resources is low. In addition, the protected area network expands. SSP3 represents the “regional rivalry” scenario in which the growth of the human population and use of resources is high. The regulation of land-use change is limited, leading to extensive deforestation. Finally, SSP5 represents the “fossil-fueled development” scenario in which the growth of the human population is low. The use of fossil fuels largely prevents the change of natural land use types into e.g. croplands for the production of biofuels (O'Neill et al., 2017; Schipper et al., 2020). In Madagascar, all three scenarios lead to a decrease in forests and shrublands in 2050, compared with 2015. The decrease is relatively small in the SSP1 scenario and largest in the SSP3 scenario (Fig. S1).

**Area of habitat calculation**

We calculated for each species the area of habitat in 2015 and for the three SSP scenarios in 2050, using the same methods as detailed in the main text. However, instead of using the IUCN habitat map to refine a species geographical range based on habitat preferences (Jung et al., 2020) we used the land-use and land cover (LULC) maps from GLOBIO (Schipper et al., 2020). This was necessary because the IUCN habitat map is currently only available for the year 2015, whereas the LULC maps from GLOBIO are available for 2015, as well as the three SSP scenarios in 2050, allowing to compare the habitat areas from 2015 and 2050. We used a cross-walk to match the IUCN habitat preferences to the GLOBIO LULC types (Gallego‐Zamorano et al., 2020).

**Comparison of time-lagged effects of habitat fragmentation with projected future habitat loss**

To compare time-lagged effects of habitat fragmentation with projected future habitat loss, we calculated for each species the habitat loss between 2015 and 2050 in the three SSP scenarios, and compared it with the habitat area hosting populations committed to extirpation.


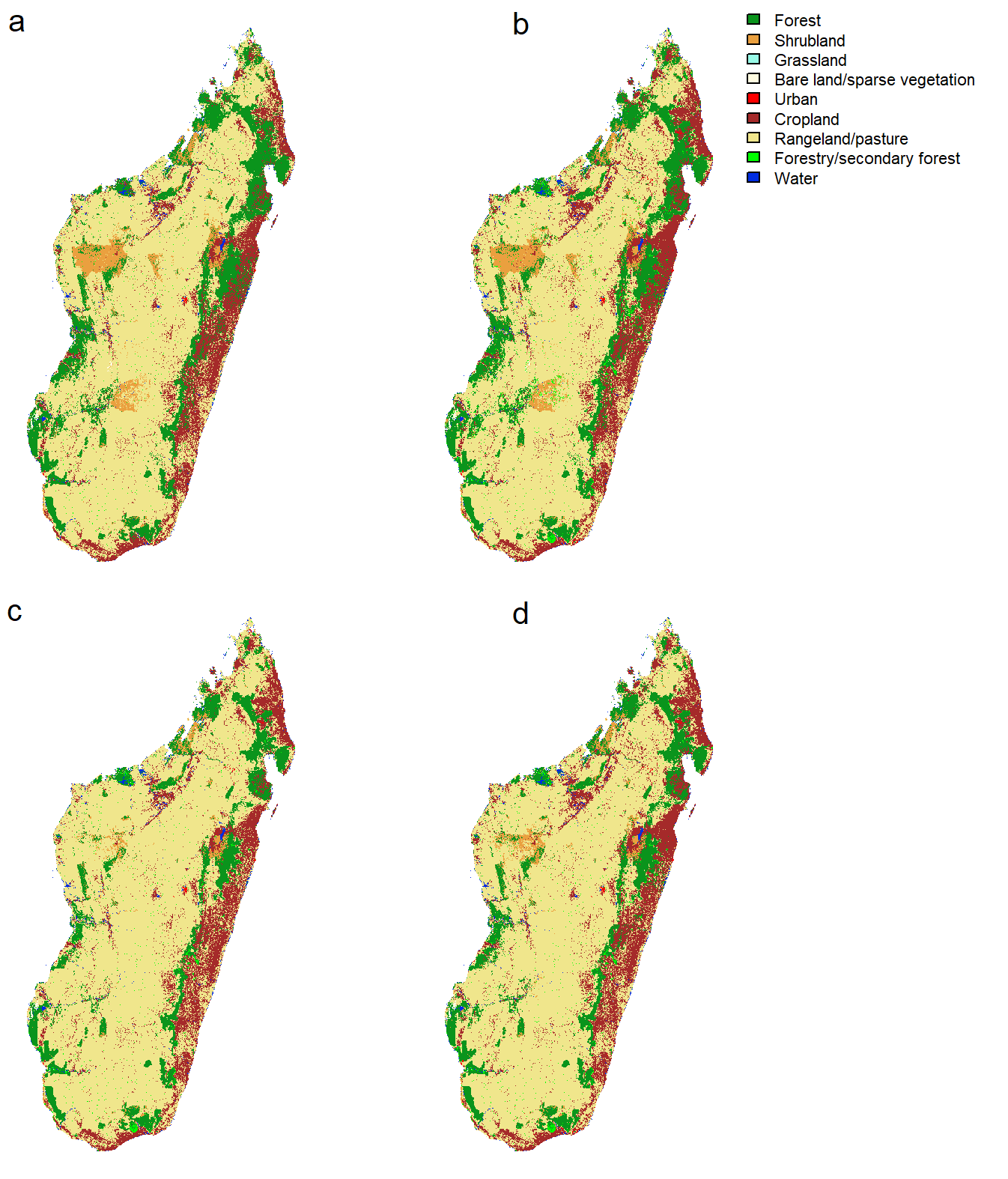


Fig. S1. Simplified land cover maps of Madagascar from GLOBIO for 2015 (a) and for 2050, based on the habitat conversion scenarios corresponding to SSP1 (b), SSP3 (c), and SSP5 (d).

LITERATURE CITED

Gallego‐Zamorano J., Benítez‐López A., Santini L., Hilbers J.P., Huijbregts M.A., Schipper A.M. (2020) Combined effects of land use and hunting on distributions of tropical mammals. *Conservation Biology*, 34, 1271-1280.

Jung M., Dahal P.R., Butchart S.H.M., Donald P.F., De Lamo X., Lesiv M., Kapos V., Rondinini C., Visconti P. (2020) A global map of terrestrial habitat types. *Scientific Data*, 7, 8.

O'Neill B.C., Kriegler E., Ebi K.L., Kemp-Benedict E., Riahi K., Rothman D.S., van Ruijven B.J., van Vuuren D.P., Birkmann J., Kok K., Levy M., Solecki W. (2017) The roads ahead: Narratives for shared socioeconomic pathways describing world futures in the 21st century. *Global Environmental Change-Human and Policy Dimensions*, 42, 169-180.

Schipper A.M., Hilbers J.P., Meijer J.R., Antao L.H., Benitez-Lopez A., de Jonge M.M.J., Leemans L.H., Scheper E., Alkemade R., Doelman J.C., Mylius S., Stehfest E., van Vuuren D.P., van Zeist W.J., Huijbregts M.A.J. (2020) Projecting terrestrial biodiversity intactness with GLOBIO 4. *Global Change Biology*, 26, 760-771.
