# Supplementary material for: Time‐lagged effects of habitat fragmentation on terrestrial mammals in Madagascar
Source: Conserv Biol. 2022 Sep 20;36(5):e13942. doi: 10.1111/cobi.13942 (PMC9826438; doi:10.1111/cobi.13942)
Supplement: Supplementary file 4 — Additional supporting information may be found in the online version of the article at the publisher's website. [file COBI-36-0-s004.docx]

APPENDIX S4. SUPPLEMENTARY RESULTS

**Results of dispersal scenario**

*Proportion of habitat area hosting populations committed to extirpation*

Based on median values for each species, we found that 1% of the species are expected to lose at least 20% of their habitat area due to time-lagged effects of habitat fragmentation (Fig. S2-S3, Appendix S3). In addition, 2% of the species are expected to lose at least 10% of their habitat area. 45%, 31%, and 11% of the species have larger time-lagged effects of habitat fragmentation than projected future habitat loss in 2050 in scenarios SSP1, SSP3, and SSP5, respectively (Appendix S3).

The results were comparable for forest specialist species (2% of the species are expected to lose at least 10% of their habitat area) and habitat generalist species (3% of the species are expected to lose at least 10% of their habitat area), but differed between species from different taxonomic orders (Fig S4). We found the largest proportions of habitat area hosting populations committed to extirpation for species from the orders Carnivora and Primates (Fig. S4).

When we studied the 0.95 quantiles instead of the median values of the proportion of habitat area hosting populations committed to extirpation, we found that 46% of the species are expected to lose at least 10% of their habitat area due to time-lagged effects of habitat fragmentation. The spread in the AOH_ex_ within each species was mostly attributed to uncertainty in the median dispersal distance (on average 56% across all species), followed by the environmental stochasticity parameter (19%), density at carrying capacity (15%), initial population density (8%), and intrinsic population growth rate (2%).

*Red List classification*

Four species (3%), including two forest specialist species, were classified to a higher Red List category when we used their habitat area hosting viable populations as the area of occupancy (AOH_100_), instead of the total initial habitat area (AOH_0_, Fig. S5). We derived these numbers using the most frequent Red List category for each species. When we followed a precautionary principle and used the highest Red List category that is reached in at least 5% of the simulations, the numbers of species with higher Red List categories changed to 41.

*Hotspots of populations committed to extirpation*

The largest absolute and relative number of populations committed to extirpation were located in eastern Madagascar (Fig. S6).


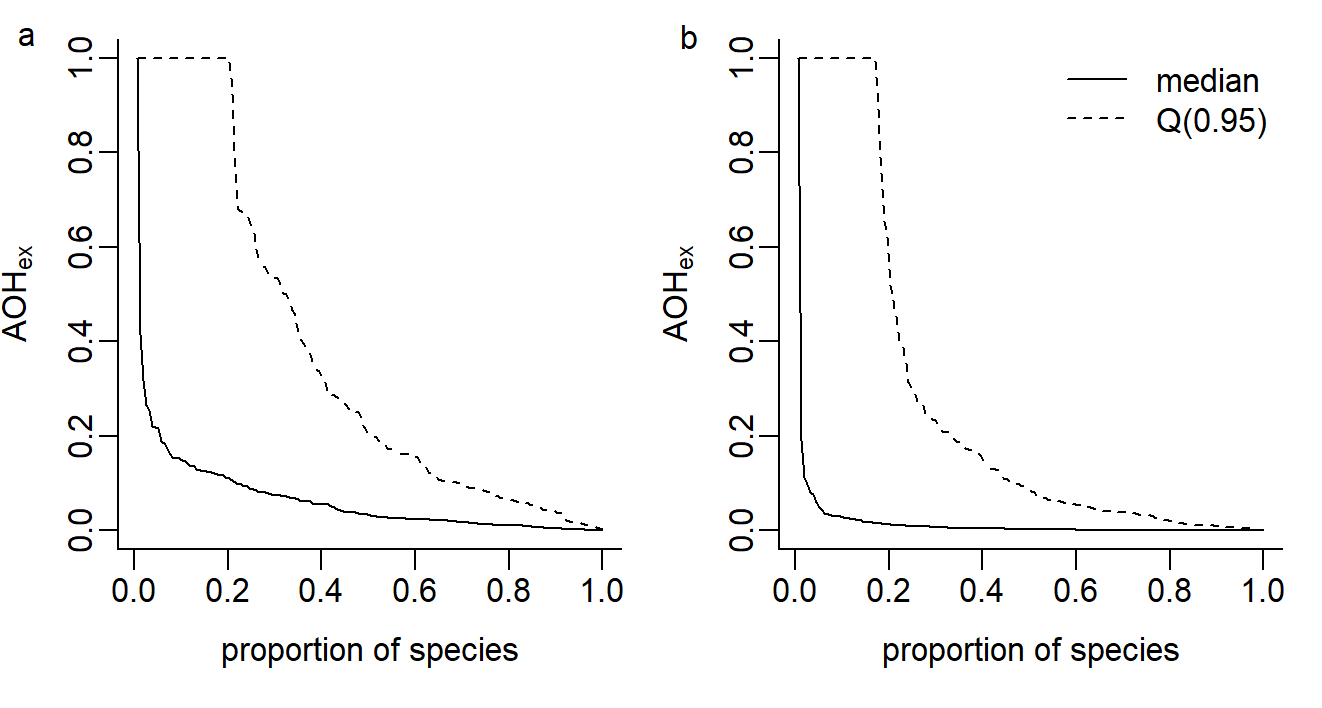


Fig. S2. Proportion of habitat area which hosts populations committed to extirpation (AOH_ex_) for each species, with species sorted based on decreasing AOH_ex_. The different panels indicate the scenario where (a) each habitat patch hosts a different population and (b) the dispersal scenario.


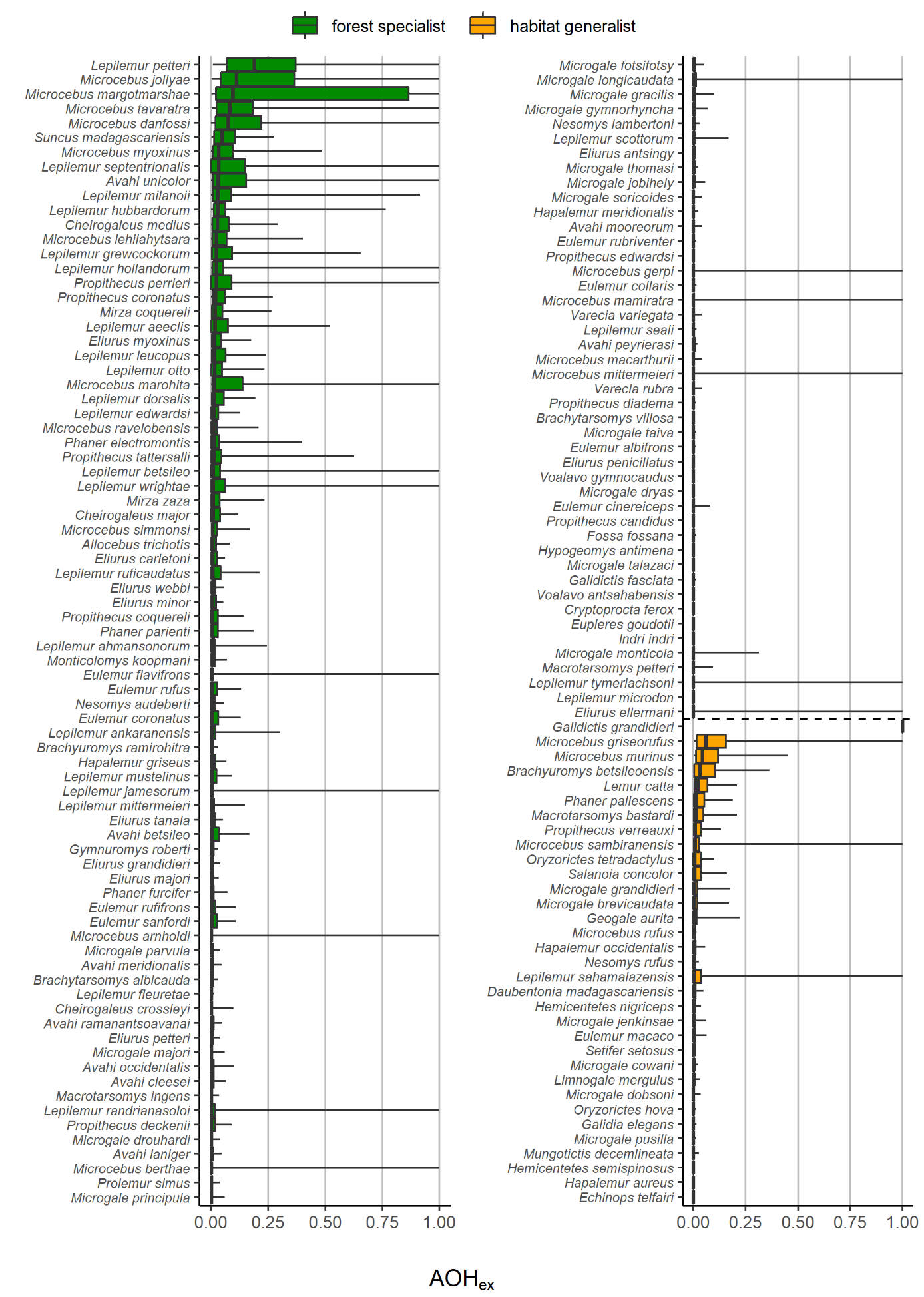


Fig. S3. Proportion of habitat area which hosts populations committed to extirpation (AOH_ex_) based on 10,000 model simulations in the dispersal scenario for forest specialist species and habitat generalists (boxes, 25th and 75th percentiles; vertical line in boxes, median; whiskers, 5th and 95th percentiles).


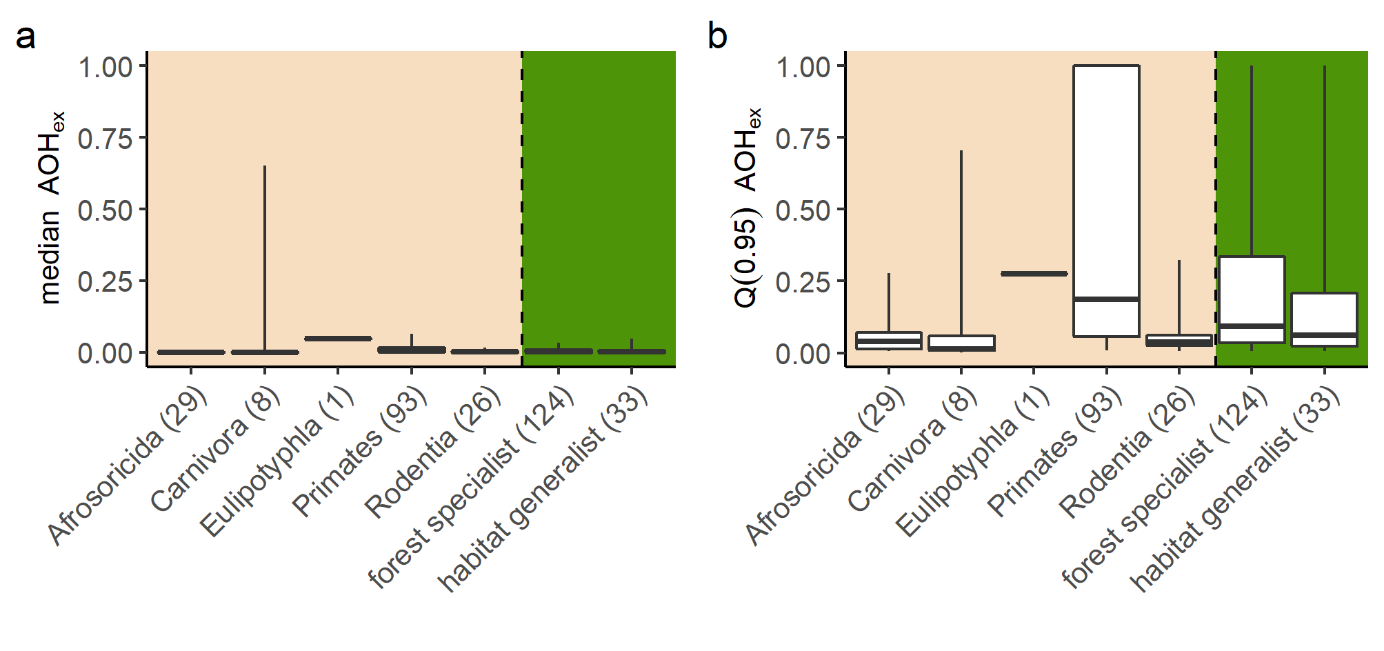


Fig. S4. Variation in the (a) median and (b) 0.95 quantile value of the proportion of habitat area which hosts populations committed to extirpation (AOH_ex_) for species from different taxonomic orders and for forest specialist and habitat generalist species in the dispersal scenario (boxes, 25th and 75th percentiles; vertical line in boxes, median; whiskers, 5th and 95th percentiles).


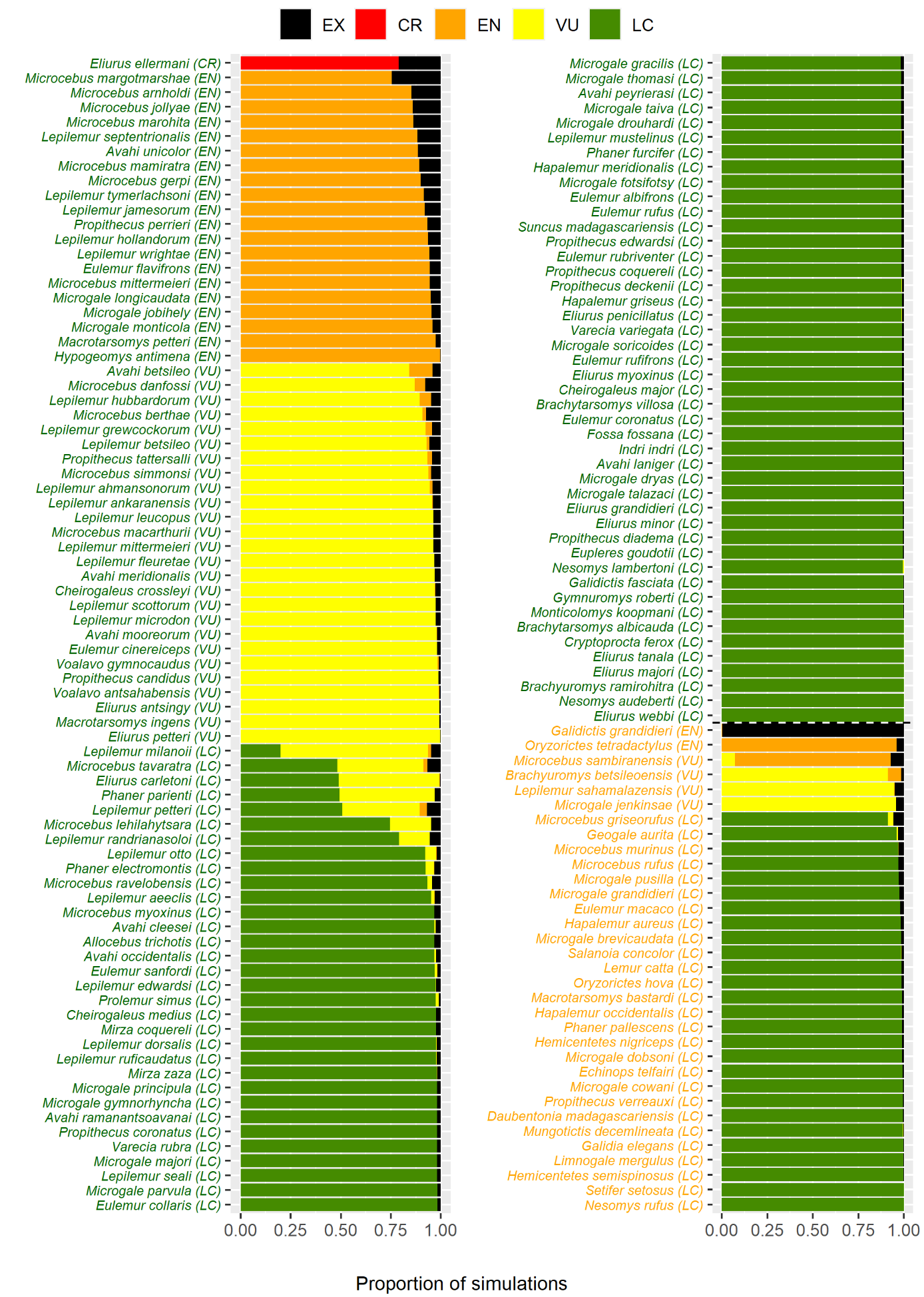


Fig. S5. Proportion of the 10,000 simulations in which a species is assigned a different International Union for the Conservation of Nature (IUCN) Red List category based on IUCN criterion B2, assuming the area of occupancy is equal to the habitat area that hosts viable populations (AOH_100_) in the dispersal scenario. Categories in parentheses indicate a species’ category based on IUCN criterion B2, assuming the area of occupancy is equal to the total habitat area (AOH_0_) (green type, forest specialist species; orange type, habitat generalist species; LC, least concern; NT, near threatened; VU, vulnerable; EN, endangered; CR, critically endangered; EX, extinct).


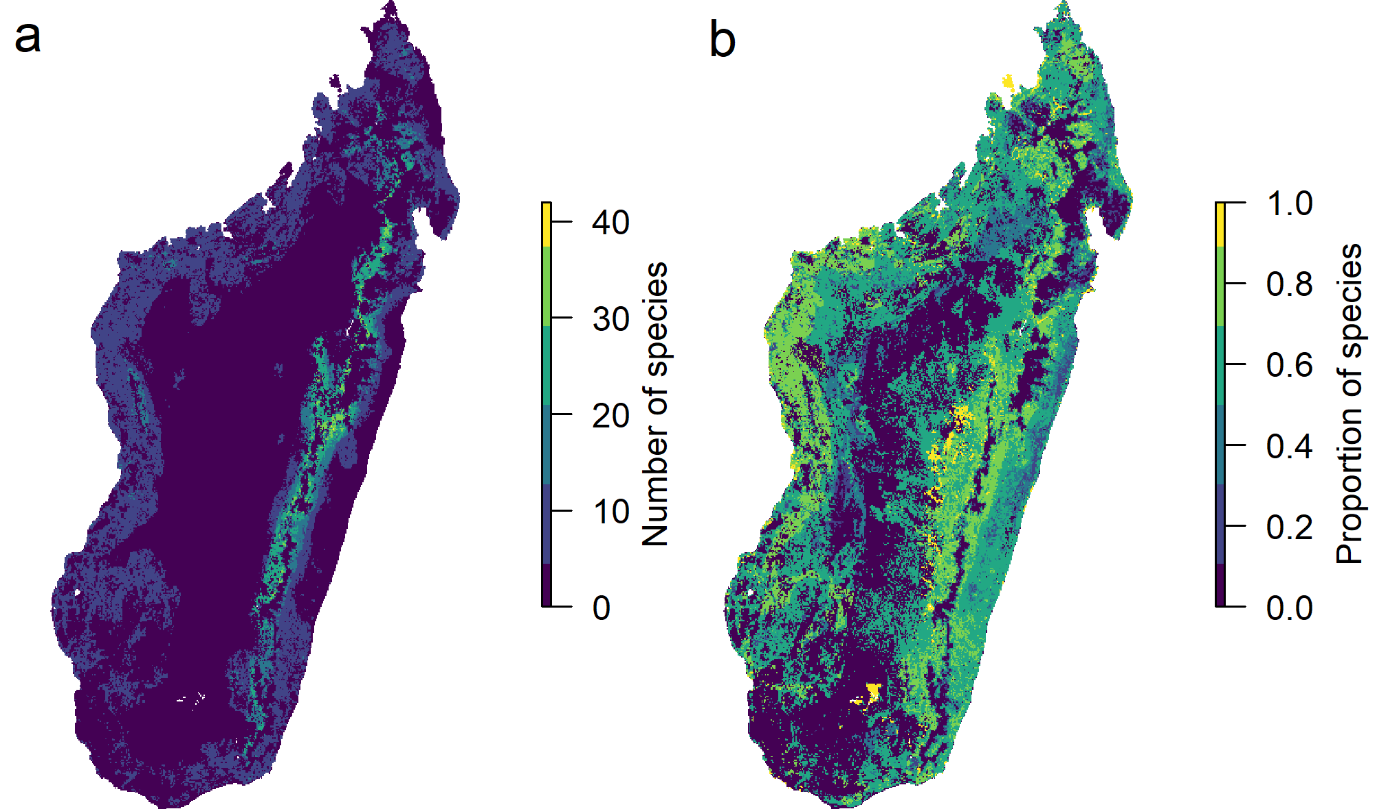


Fig. S6: In Madagascar (a) absolute and (b) relative number of species committed to extirpation (probability of extinction within 100 years >5%) in the dispersal scenario.
