# Supplementary material for: Time‐lagged effects of habitat fragmentation on terrestrial mammals in Madagascar
Source: Conserv Biol. 2022 Sep 20;36(5):e13942. doi: 10.1111/cobi.13942 (PMC9826438; doi:10.1111/cobi.13942)
Supplement: Supplementary file 5 — Additional supporting information may be found in the online version of the article at the publisher's website. [file COBI-36-0-s001.docx]

APPENDIX S5. SENSITIVITY TO ALLOMETRICALLY DERIVED PARAMETERS

To estimate the sensitivity of the results to using values of the population parameters estimated from allometric relationships (hereafter allometric analyses) instead of using empirical estimates, we also ran the analyses using empirical estimates of the population parameters (hereafter empirical analyses). Empirical estimates for the median dispersal distance, intrinsic population growth rate and population density could be obtained from the literature for 24 species of Malagasy mammals (15% of all species in the study, see Table S4 for parameter values). Because population densities can vary both spatially and temporally, we only included empirical values of the population density for species when there were 10 or more population density estimates found. For species with a lower number of density estimates, it is difficult to establish whether the population densities represent a maximum density value (population density at carrying capacity) or a lower population density. The initial population density was then calculated as the median density value, and the population density at carrying capacity was calculated as the 0.95 quantile value of the densities (Table S5). For the grey mouse lemur (*Microcebus murinus*) separate median dispersal distances were found for males and females. In our analyses we used the average of these values (Table S5).

For none of the species we found empirical estimates for all of the population parameters. In the no-dispersal scenario, estimates of four parameters were required for each species (intrinsic population growth rate, environmental stochasticity, initial population density, and population density at carrying capacity), but for each species we found empirical values for only 1-3 parameters (Table S5). This leads to a total of 56% of the parameter values across all 24 species for which we could use empirical estimates. In the dispersal scenario, we also needed an estimate of the median dispersal distance, which was only available for the grey mouse lemur. For this species, we could use empirical values for four of the five parameters (80%) in the dispersal scenario. For the population parameters for which empirical estimates could not be found, we randomly sampled values using allometric relationships, similar to the allometric analyses.

We ran the empirical analyses for 24 species in the no-dispersal scenario and for one species in the dispersal scenario. We ran the empirical analyses of the dispersal scenario only for the grey mouse lemur, because it is the only species for which we obtained an empirical estimate of the median dispersal distance. Similar to allometric analyses (described in the main text), we ran 10,000 simulations for the empirical analyses to calculate time-lagged effects of habitat fragmentation and assigned species to IUCN Red List categories according to criterion B2.

To compare the AOH_ex_ (proportion of habitat area hosting populations committed to extirpation) between the two analyses, we plotted kernel density plots of the AOH_ex_ for both analyses and calculated the proportion of the distributions that overlapped. High proportional overlap of these distributions indicate comparable results for the allometric and empirical analyses. A low proportional overlap indicates differences in the distribution of AOH_ex_ values between these two analyses, but this does not necessarily imply that the results from the allometric analyses are biased. For example, in the empirical analyses, the parameters for which we had empirical values were not variable between the simulations (contrary to the allometric analyses), which decreased the uncertainty in AOH_ex_ values. This could lead to a narrower peak of the kernel density distribution of AOH_ex_ values from the empirical analyses, relative to allometric analyses, and hence kernel density distributions that are partly non-overlapping. Therefore, to check whether the use of allometric relationships to estimate parameter values biased our results, we also calculated the proportion of AOH_ex_ values from the empirical analyses for each species that fell within the range of values from the allometric analyses. To compare the classifications into IUCN Red List categories according to criterion B2, we visually compared the number of simulations each species was classified into different IUCN Red List categories.

For the 24 species for which we had empirical parameter estimates, all the empirical parameter estimates fell within the 90% confidence interval of the parameter estimates from allometric relationships (Table S4). The overlap in the kernel density distributions of AOH_ex_ values from the allometric and empirical analyses varied between 0.39-0.91 with an average of 0.58 for the 24 species in the no-dispersal scenario (Fig. S7). In the dispersal scenario, the overlap in AOH_ex_ values was 0.71 for the grey mouse lemur (Fig. S8). On average, the distributions of AOH_ex_ from the empirical and allometric analyses were thus comparable and also visually the kernel distributions of the empirical and allometric analyses looked similar (Fig. S7-S8). In addition, even though for some species (e.g. *Lemur catta*, *Varecia variegata*) the proportional overlap of the distributions was relatively low, the proportion of AOH_ex_ values from the empirical analyses that fell within the range of values from the allometric analyses was larger than 0.92 for all species (Fig. S7-S8). The relatively low proportional of overlap for some species is thus caused by the lower uncertainty in AOH_ex_ in the empirical analyses, leading to narrower peaks. Finally, the proportions of the simulations in which each species was classified into different IUCN Red List categories were also largely similar between the allometric and empirical analyses (Fig. S9-S10). The results from the sensitivity analysis thus indicates that our results were not changed considerably by using allometric relationships to estimate parameter values.

Table S4. Empirical estimates of the median dispersal distance, intrinsic population growth rate, and population density for a subset of Malagasy mammal species.

| Species | Parameter^*^ | Value | Reference |
| --- | --- | --- | --- |
| *Microcebus murinus* | Dispersal (males) | 0.251 | (Radespiel et al., 2003) |
| *Microcebus murinus* | Dispersal (females) | 0.063 | (Radespiel et al., 2003) |
| *Cheirogaleus medius* | r | 0.64 | (Ross, 1992) |
| *Daubentonia madagascariensis* | r | 0.15 | (Ross, 1988) |
| *Eulemur fulvus* | r | 0.23 | (Ross, 1992) |
| *Eulemur macaco* | r | 0.30 | (Ross, 1992) |
| *Lemur catta* | r | 0.27 | (Ross, 1992) |
| *Microcebus murinus* | r | 0.67 | (Ross, 1992) |
| *Mirza coquereli* | r | 0.56 | (Ross, 1992) |
| *Propithecus verreauxi* | r | 0.27 | (Ross, 1988) |
| *Varecia variegata* | r | 0.46 | (Ross, 1992) |
| *Avahi laniger* | Density | 10 | (Sterling & McFadden, 2000) |
| *Avahi laniger* | Density | 17.85 | (Rasolofoson et al., 2007) |
| *Avahi laniger* | Density | 18 | (Rasolofoson et al., 2007) |
| *Avahi laniger* | Density | 20.3 | (Hawkins et al., 2005) |
| *Avahi laniger* | Density | 20.7 | (Rasolofoson et al., 2007) |
| *Avahi laniger* | Density | 25 | (Sterling & Rakotoarison, 1998) |
| *Avahi laniger* | Density | 31.36 | (Rasolofoson et al., 2007) |
| *Avahi laniger* | Density | 37 | (Rasolofoson et al., 2007) |
| *Avahi laniger* | Density | 43 | (Rasolofoson et al., 2007) |
| *Avahi laniger* | Density | 44 | (Ralison, 2006b) |
| *Avahi laniger* | Density | 55 | (Rasolofoson et al., 2007) |
| *Avahi laniger* | Density | 57.7 | (Murphy et al., 2016) |
| *Avahi laniger* | Density | 63 | (Ralison et al., 2015) |
| *Avahi laniger* | Density | 67 | (Ganzhorn, 1988) |
| *Avahi laniger* | Density | 72 | (Ganzhorn, 1988) |
| *Avahi laniger* | Density | 72 | (Ganzhorn, 1992) |
| *Avahi laniger* | Density | 74 | (Rasolofoson et al., 2007) |
| *Avahi laniger* | Density | 79 | (Ralison et al., 2015) |
| *Avahi laniger* | Density | 83 | (Rasolofoson et al., 2007) |
| *Avahi laniger* | Density | 89 | (Rasolofoson et al., 2007) |
| *Avahi laniger* | Density | 92 | (Rasolofoson et al., 2007) |
| *Avahi laniger* | Density | 115 | (Ralison et al., 2015) |
| *Avahi laniger* | Density | 125 | (Ralison, 2006b) |
| *Cheirogaleus major* | Density | 4.6 | (Rasolofoson et al., 2007) |
| *Cheirogaleus major* | Density | 8.4 | (Rasolofoson et al., 2007) |
| *Cheirogaleus major* | Density | 9.5 | (Rasolofoson et al., 2007) |
| *Cheirogaleus major* | Density | 25 | (Ralison, 2006b) |
| *Cheirogaleus major* | Density | 27 | (Rasolofoson et al., 2007) |
| *Cheirogaleus major* | Density | 28 | (Rasolofoson et al., 2007) |
| *Cheirogaleus major* | Density | 33 | (Ralison, 2006a) |
| *Cheirogaleus major* | Density | 38 | (Rasolofoson et al., 2007) |
| *Cheirogaleus major* | Density | 42 | (Rasolofoson et al., 2007) |
| *Cheirogaleus major* | Density | 47 | (Sterling & Rakotoarison, 1998) |
| *Cheirogaleus major* | Density | 48.7 | (Hawkins et al., 2005) |
| *Cheirogaleus major* | Density | 49 | (Rasolofoson et al., 2007) |
| *Cheirogaleus major* | Density | 67 | (Ralison et al., 2015) |
| *Cheirogaleus major* | Density | 68 | (Ganzhorn, 1992) |
| *Cheirogaleus major* | Density | 104 | (Rasolofoson et al., 2007) |
| *Cheirogaleus major* | Density | 104 | (Rasolofoson et al., 2007) |
| *Cheirogaleus major* | Density | 131 | (Ralison et al., 2015) |
| *Cheirogaleus medius* | Density | 20 | (Hladik et al., 1980) |
| *Cheirogaleus medius* | Density | 37 | (Jolly, 1985) |
| *Cheirogaleus medius* | Density | 37 | (Jolly, 1986) |
| *Cheirogaleus medius* | Density | 46 | (Ralison, 2008) |
| *Cheirogaleus medius* | Density | 50 | (Ralison, 2007) |
| *Cheirogaleus medius* | Density | 50 | (Ralison, 2008) |
| *Cheirogaleus medius* | Density | 74 | (Ralison, 2008) |
| *Cheirogaleus medius* | Density | 79 | (Ralison, 2008) |
| *Cheirogaleus medius* | Density | 81 | (Ganzhorn, 1988) |
| *Cheirogaleus medius* | Density | 81 | (Ausilio & Raveloanrinoro, 1998) |
| *Cheirogaleus medius* | Density | 83 | (Ralison, 2008) |
| *Cheirogaleus medius* | Density | 90 | (Schäffler & Kappeler, 2014b) |
| *Cheirogaleus medius* | Density | 99 | (Ausilio & Raveloanrinoro, 1998) |
| *Cheirogaleus medius* | Density | 100 | (Ralison, 2008) |
| *Cheirogaleus medius* | Density | 139 | (Schäffler & Kappeler, 2014b) |
| *Cheirogaleus medius* | Density | 180 | (Schäffler & Kappeler, 2014b) |
| *Cheirogaleus medius* | Density | 200 | (Hladik et al., 1980) |
| *Cheirogaleus medius* | Density | 202 | (Ralison, 2008) |
| *Cheirogaleus medius* | Density | 203 | (Ralison, 2008) |
| *Cheirogaleus medius* | Density | 220 | (Ralison, 2008) |
| *Cheirogaleus medius* | Density | 238 | (Ralison, 2008) |
| *Cheirogaleus medius* | Density | 240 | (Reed, 1999) |
| *Cheirogaleus medius* | Density | 258 | (Schäffler & Kappeler, 2014b) |
| *Cheirogaleus medius* | Density | 280 | (Fietz, 1999) |
| *Cheirogaleus medius* | Density | 293 | (Ralison, 2008) |
| *Cheirogaleus medius* | Density | 350 | (Hladik et al., 1980) |
| *Cheirogaleus medius* | Density | 360 | (Fietz, 1999) |
| *Cheirogaleus medius* | Density | 566 | (Ralison, 2008) |
| *Cheirogaleus medius* | Density | 750 | (Ralison, 2008) |
| *Eulemur albifrons* | Density | 0.4 | (Rasolofoson et al., 2007) |
| *Eulemur albifrons* | Density | 4.1 | (Rasolofoson et al., 2007) |
| *Eulemur albifrons* | Density | 4.62 | (Rasolofoson et al., 2007) |
| *Eulemur albifrons* | Density | 5 | (Sterling & McFadden, 2000) |
| *Eulemur albifrons* | Density | 5.74 | (Rasolofoson et al., 2007) |
| *Eulemur albifrons* | Density | 9.45 | (Rasolofoson et al., 2007) |
| *Eulemur albifrons* | Density | 15 | (Sterling & McFadden, 2000) |
| *Eulemur albifrons* | Density | 21.5 | (Murphy et al., 2016) |
| *Eulemur albifrons* | Density | 29.1 | (Rasolofoson et al., 2007) |
| *Eulemur albifrons* | Density | 31.8 | (Rasolofoson et al., 2007) |
| *Eulemur albifrons* | Density | 38.2 | (Rasolofoson et al., 2007) |
| *Eulemur albifrons* | Density | 38.8 | (Rasolofoson et al., 2007) |
| *Eulemur albifrons* | Density | 47 | (Sterling & McFadden, 2000) |
| *Eulemur albifrons* | Density | 48.3 | (Rasolofoson et al., 2007) |
| *Eulemur albifrons* | Density | 50.3 | (Rasolofoson et al., 2007) |
| *Eulemur albifrons* | Density | 75 | (Ralison, 2006b) |
| *Eulemur albifrons* | Density | 82.2 | (Rasolofoson et al., 2007) |
| *Eulemur albifrons* | Density | 136 | (Ralison, 2006b) |
| *Eulemur albifrons* | Density | 199 | (Sterling & McFadden, 2000) |
| *Eulemur collaris* | Density | 1 | (Rasoarimanana, 2005) |
| *Eulemur collaris* | Density | 1 | (Rasoarimanana, 2005) |
| *Eulemur collaris* | Density | 1 | (Rasoarimanana, 2005) |
| *Eulemur collaris* | Density | 3 | (Rasoarimanana, 2005) |
| *Eulemur collaris* | Density | 5 | (Rasoarimanana, 2005) |
| *Eulemur collaris* | Density | 8 | (Rasoarimanana, 2005) |
| *Eulemur collaris* | Density | 8 | (Rasoarimanana, 2005) |
| *Eulemur collaris* | Density | 8 | (Rasoarimanana, 2005) |
| *Eulemur collaris* | Density | 8 | (Rasoarimanana, 2005) |
| *Eulemur collaris* | Density | 10 | (Rasoarimanana, 2005) |
| *Eulemur collaris* | Density | 10 | (Rasoarimanana, 2005) |
| *Eulemur collaris* | Density | 10 | (Rasoarimanana, 2005) |
| *Eulemur collaris* | Density | 10.7 | (Johnson & Overdorff, 1999) |
| *Eulemur collaris* | Density | 12 | (Rasoarimanana, 2005) |
| *Eulemur collaris* | Density | 12 | (Rasoarimanana, 2005) |
| *Eulemur collaris* | Density | 23 | (Rasoarimanana, 2005) |
| *Eulemur collaris* | Density | 28 | (Rasoarimanana, 2005) |
| *Eulemur collaris* | Density | 30 | (Rasoarimanana, 2005) |
| *Eulemur rubriventer* | Density | 0.52 | (Rakotosamimanana et al., 2004) |
| *Eulemur rubriventer* | Density | 0.62 | (Rasolofoson et al., 2007) |
| *Eulemur rubriventer* | Density | 3 | (Ralison et al., 2015) |
| *Eulemur rubriventer* | Density | 3.48 | (Rakotosamimanana et al., 2004) |
| *Eulemur rubriventer* | Density | 4.08 | (Rasolofoson et al., 2007) |
| *Eulemur rubriventer* | Density | 4.46 | (Rasolofoson et al., 2007) |
| *Eulemur rubriventer* | Density | 6.28 | (Rakotosamimanana et al., 2004) |
| *Eulemur rubriventer* | Density | 8.93 | (Rasolofoson et al., 2007) |
| *Eulemur rubriventer* | Density | 10 | (Ralison, 2006b) |
| *Eulemur rubriventer* | Density | 11 | (Ralison et al., 2015) |
| *Eulemur rubriventer* | Density | 12.7 | (Rasolofoson et al., 2007) |
| *Eulemur rubriventer* | Density | 15 | (Ralison et al., 2015) |
| *Eulemur rubriventer* | Density | 15.3 | (Rasolofoson et al., 2007) |
| *Eulemur rubriventer* | Density | 20.9 | (Hawkins et al., 2005) |
| *Eulemur rubriventer* | Density | 23.5 | (Rasolofoson et al., 2007) |
| *Eulemur rubriventer* | Density | 30 | (Overdorff, 1988) |
| *Eulemur rubriventer* | Density | 30 | (Ganzhorn, 1992) |
| *Eulemur rubriventer* | Density | 37 | (Sterling & McFadden, 2000) |
| *Eulemur rubriventer* | Density | 40 | (Sterling & McFadden, 2000) |
| *Eulemur rubriventer* | Density | 48.6 | (Rasolofoson et al., 2007) |
| *Eulemur rufus* | Density | 3.8 | (Hawkins et al., 2005) |
| *Eulemur rufus* | Density | 11.4 | (Johnson & Overdorff, 1999) |
| *Eulemur rufus* | Density | 13 | (Johnson & Overdorff, 1999) |
| *Eulemur rufus* | Density | 15 | (Ralison, 2007) |
| *Eulemur rufus* | Density | 22 | (Ganzhorn, 1992) |
| *Eulemur rufus* | Density | 70 | (Ganzhorn, 1992) |
| *Eulemur rufus* | Density | 103 | (Ganzhorn, 1992) |
| *Eulemur rufus* | Density | 121.4 | (Müller et al., 2000) |
| *Eulemur rufus* | Density | 137 | (Ausilio & Raveloanrinoro, 1998) |
| *Eulemur rufus* | Density | 213 | (Ausilio & Raveloanrinoro, 1998) |
| *Hapalemur griseus* | Density | 0.15 | (Rasolofoson et al., 2007) |
| *Hapalemur griseus* | Density | 0.48 | (Rasolofoson et al., 2007) |
| *Hapalemur griseus* | Density | 0.81 | (Rasolofoson et al., 2007) |
| *Hapalemur griseus* | Density | 0.99 | (Rasolofoson et al., 2007) |
| *Hapalemur griseus* | Density | 1.04 | (Rasolofoson et al., 2007) |
| *Hapalemur griseus* | Density | 1.30 | (Rasolofoson et al., 2007) |
| *Hapalemur griseus* | Density | 2.13 | (Rasolofoson et al., 2007) |
| *Hapalemur griseus* | Density | 3 | (Rasolofoson et al., 2007) |
| *Hapalemur griseus* | Density | 3.05 | (Rakotosamimanana et al., 2004) |
| *Hapalemur griseus* | Density | 5.84 | (Rasolofoson et al., 2007) |
| *Hapalemur griseus* | Density | 6 | (Sterling & Rakotoarison, 1998) |
| *Hapalemur griseus* | Density | 6.8 | (Rasolofoson et al., 2007) |
| *Hapalemur griseus* | Density | 7 | (Rasoarimanana, 2005) |
| *Hapalemur griseus* | Density | 7.2 | (Hawkins et al., 2005) |
| *Hapalemur griseus* | Density | 7.91 | (Rakotosamimanana et al., 2004) |
| *Hapalemur griseus* | Density | 8.75 | (Rakotosamimanana et al., 2004) |
| *Hapalemur griseus* | Density | 13 | (Ralison, 2006b) |
| *Hapalemur griseus* | Density | 16 | (Ausilio & Raveloanrinoro, 1998) |
| *Hapalemur griseus* | Density | 24 | (Sterling & McFadden, 2000) |
| *Hapalemur griseus* | Density | 44 | (Ralison et al., 2015) |
| *Hapalemur griseus* | Density | 46 | (Ralison et al., 2015) |
| *Hapalemur griseus* | Density | 55 | (Ganzhorn, 1992) |
| *Hapalemur griseus* | Density | 59 | (Ralison et al., 2015) |
| *Hapalemur griseus* | Density | 69.5 | (Sterling & McFadden, 2000) |
| *Indri indri* | Density | 1.31 | (Rasolofoson et al., 2007) |
| *Indri indri* | Density | 1.47 | (Rasolofoson et al., 2007) |
| *Indri indri* | Density | 1.63 | (Rasolofoson et al., 2007) |
| *Indri indri* | Density | 2.1 | (Rasolofoson et al., 2007) |
| *Indri indri* | Density | 2.9 | (Glessner & Britt, 2005) |
| *Indri indri* | Density | 3.32 | (Rakotosamimanana et al., 2004) |
| *Indri indri* | Density | 5.6 | (Glessner & Britt, 2005) |
| *Indri indri* | Density | 5.62 | (Rasolofoson et al., 2007) |
| *Indri indri* | Density | 6.6 | (Glessner & Britt, 2005) |
| *Indri indri* | Density | 6.7 | (Rasolofoson et al., 2007) |
| *Indri indri* | Density | 6.9 | (Glessner & Britt, 2005) |
| *Indri indri* | Density | 7.45 | (Rasolofoson et al., 2007) |
| *Indri indri* | Density | 7.57 | (Rakotosamimanana et al., 2004) |
| *Indri indri* | Density | 8 | (Glessner & Britt, 2005) |
| *Indri indri* | Density | 8.3 | (Rakotosamimanana et al., 2004) |
| *Indri indri* | Density | 11 | (Ralison et al., 2015) |
| *Indri indri* | Density | 12 | (Ralison et al., 2015) |
| *Indri indri* | Density | 12.5 | (Ganzhorn, 1992) |
| *Indri indri* | Density | 13.2 | (Glessner & Britt, 2005) |
| *Indri indri* | Density | 14 | (Ralison, 2006b) |
| *Indri indri* | Density | 15.5 | (Rasolofoson et al., 2007) |
| *Indri indri* | Density | 16 | (Glessner & Britt, 2005) |
| *Indri indri* | Density | 17.5 | (Rasolofoson et al., 2007) |
| *Indri indri* | Density | 23 | (Ralison et al., 2015) |
| *Lemur catta* | Density | 5 | (Rasoarimanana, 2005) |
| *Lemur catta* | Density | 8 | (Rasoarimanana, 2005) |
| *Lemur catta* | Density | 10 | (Rasoarimanana, 2005) |
| *Lemur catta* | Density | 10 | (Rasoarimanana, 2005) |
| *Lemur catta* | Density | 18 | (Rasoarimanana, 2005) |
| *Lemur catta* | Density | 20 | (Rasoarimanana, 2005) |
| *Lemur catta* | Density | 25 | (Rasoarimanana, 2005) |
| *Lemur catta* | Density | 25 | (Rasoarimanana, 2005) |
| *Lemur catta* | Density | 25 | (Rasoarimanana, 2005) |
| *Lemur catta* | Density | 30 | (Rasoarimanana, 2005) |
| *Lemur catta* | Density | 30 | (Rasoarimanana, 2005) |
| *Lemur catta* | Density | 30 | (Rasoarimanana, 2005) |
| *Lemur catta* | Density | 36 | (Rasoarimanana, 2005) |
| *Lemur catta* | Density | 38 | (Rasoarimanana, 2005) |
| *Lemur catta* | Density | 60 | (Rasoarimanana, 2005) |
| *Lemur catta* | Density | 60 | (Rasoarimanana, 2005) |
| *Lemur catta* | Density | 70 | (Rasoarimanana, 2005) |
| *Lemur catta* | Density | 99.75 | (Gould et al., 1999) |
| *Lemur catta* | Density | 102.25 | (Gould et al., 1999) |
| *Lemur catta* | Density | 102.25 | (Gould et al., 1999) |
| *Lemur catta* | Density | 105 | (Jolly et al., 2002) |
| *Lemur catta* | Density | 107.25 | (Gould et al., 1999) |
| *Lemur catta* | Density | 109 | (Jolly et al., 2002) |
| *Lemur catta* | Density | 117.25 | (Gould et al., 1999) |
| *Lemur catta* | Density | 123.5 | (Gould et al., 1999) |
| *Lemur catta* | Density | 123.5 | (Gould et al., 1999) |
| *Lemur catta* | Density | 125 | (Jolly et al., 2002) |
| *Lemur catta* | Density | 129.75 | (Gould et al., 1999) |
| *Lemur catta* | Density | 138.5 | (Gould et al., 1999) |
| *Lemur catta* | Density | 142.25 | (Gould et al., 1999) |
| *Lemur catta* | Density | 153 | (Jolly et al., 2002) |
| *Lemur catta* | Density | 155 | (Jolly et al., 2002) |
| *Lemur catta* | Density | 226 | (Jolly et al., 2002) |
| *Lemur catta* | Density | 236 | (Jolly et al., 2002) |
| *Lemur catta* | Density | 236 | (Jolly et al., 2002) |
| *Lemur catta* | Density | 243 | (Jolly et al., 2002) |
| *Lemur catta* | Density | 262 | (Jolly et al., 2002) |
| *Lemur catta* | Density | 264 | (Jolly et al., 2002) |
| *Lemur catta* | Density | 266 | (Jolly et al., 2002) |
| *Lemur catta* | Density | 282 | (Jolly et al., 2002) |
| *Lepilemur mustelinus* | Density | 7.1 | (Rasolofoson et al., 2007) |
| *Lepilemur mustelinus* | Density | 13 | (Ganzhorn, 1988) |
| *Lepilemur mustelinus* | Density | 14 | (Rasolofoson et al., 2007) |
| *Lepilemur mustelinus* | Density | 15 | (Rasolofoson et al., 2007) |
| *Lepilemur mustelinus* | Density | 15 | (Rasolofoson et al., 2007) |
| *Lepilemur mustelinus* | Density | 18 | (Sterling & McFadden, 2000) |
| *Lepilemur mustelinus* | Density | 18 | (Sterling & McFadden, 2000) |
| *Lepilemur mustelinus* | Density | 32 | (Rasolofoson et al., 2007) |
| *Lepilemur mustelinus* | Density | 33 | (Sterling & Rakotoarison, 1998) |
| *Lepilemur mustelinus* | Density | 34 | (Rasolofoson et al., 2007) |
| *Lepilemur mustelinus* | Density | 39 | (Ralison et al., 2015) |
| *Lepilemur mustelinus* | Density | 39.6 | (Rasolofoson et al., 2007) |
| *Lepilemur mustelinus* | Density | 46 | (Rasolofoson et al., 2007) |
| *Lepilemur mustelinus* | Density | 57 | (Ganzhorn, 1988) |
| *Lepilemur mustelinus* | Density | 75 | (Ralison, 2006b) |
| *Lepilemur mustelinus* | Density | 76.3 | (Rasolofoson et al., 2007) |
| *Lepilemur mustelinus* | Density | 89 | (Rasolofoson et al., 2007) |
| *Lepilemur mustelinus* | Density | 99 | (Ralison et al., 2015) |
| *Lepilemur mustelinus* | Density | 120 | (Ralison et al., 2015) |
| *Lepilemur mustelinus* | Density | 126 | (Rasolofoson et al., 2007) |
| *Microcebus berthae* | Density | 34 | (Schäffler & Kappeler, 2014a) |
| *Microcebus berthae* | Density | 55 | (Schäffler & Kappeler, 2014a) |
| *Microcebus berthae* | Density | 57 | (Schäffler & Kappeler, 2014a) |
| *Microcebus berthae* | Density | 69 | (Schäffler & Kappeler, 2014a) |
| *Microcebus berthae* | Density | 80 | (Schwab & Ganzhorn, 2004) |
| *Microcebus berthae* | Density | 95 | (Schäffler & Kappeler, 2014a) |
| *Microcebus berthae* | Density | 119 | (Schwab & Ganzhorn, 2004) |
| *Microcebus berthae* | Density | 181 | (Schäffler & Kappeler, 2014a) |
| *Microcebus berthae* | Density | 233 | (Ralison, 2008) |
| *Microcebus berthae* | Density | 327 | (Ralison, 2008) |
| *Microcebus berthae* | Density | 352 | (Ralison, 2008) |
| *Microcebus berthae* | Density | 360 | (Ralison, 2008) |
| *Microcebus berthae* | Density | 1272 | (Ralison, 2008) |
| *Microcebus danfossi* | Density | 220 | (Randrianambinina et al., 2010) |
| *Microcebus danfossi* | Density | 270 | (Randrianambinina et al., 2010) |
| *Microcebus danfossi* | Density | 340 | (Randrianambinina et al., 2010) |
| *Microcebus danfossi* | Density | 350 | (Randrianambinina et al., 2010) |
| *Microcebus danfossi* | Density | 360 | (Randrianambinina et al., 2010) |
| *Microcebus danfossi* | Density | 370 | (Randrianambinina et al., 2010) |
| *Microcebus danfossi* | Density | 410 | (Randrianambinina et al., 2010) |
| *Microcebus danfossi* | Density | 430 | (Randrianambinina et al., 2010) |
| *Microcebus danfossi* | Density | 460 | (Randrianambinina et al., 2010) |
| *Microcebus danfossi* | Density | 480 | (Randrianambinina et al., 2010) |
| *Microcebus danfossi* | Density | 500 | (Randrianambinina et al., 2010) |
| *Microcebus griseorufus* | Density | 220 | (Génin, 2008) |
| *Microcebus griseorufus* | Density | 220 | (Blanco et al., 2015) |
| *Microcebus griseorufus* | Density | 250 | (Blanco et al., 2015) |
| *Microcebus griseorufus* | Density | 273 | (Ralison, 2006b) |
| *Microcebus griseorufus* | Density | 273 | (Ralison, 2008) |
| *Microcebus griseorufus* | Density | 300 | (Ralison, 2006b) |
| *Microcebus griseorufus* | Density | 300 | (Ralison, 2008) |
| *Microcebus griseorufus* | Density | 340 | (Blanco et al., 2015) |
| *Microcebus griseorufus* | Density | 400 | (Bohr et al., 2011) |
| *Microcebus griseorufus* | Density | 546 | (Gardner et al., 2009) |
| *Microcebus griseorufus* | Density | 1000 | (Ralison, 2006b) |
| *Microcebus griseorufus* | Density | 1000 | (Ralison, 2008) |
| *Microcebus griseorufus* | Density | 1078 | (Gardner et al., 2009) |
| *Microcebus griseorufus* | Density | 1180 | (Bohr et al., 2011) |
| *Microcebus murinus* | Density | 3 | (Ganzhorn, 1992) |
| *Microcebus murinus* | Density | 40 | (Schwab & Ganzhorn, 2004) |
| *Microcebus murinus* | Density | 42 | (Ganzhorn, 1992) |
| *Microcebus murinus* | Density | 80 | (Schwab & Ganzhorn, 2004) |
| *Microcebus murinus* | Density | 85.4 | (Müller et al., 2000) |
| *Microcebus murinus* | Density | 97 | (Schwab & Ganzhorn, 2004) |
| *Microcebus murinus* | Density | 100 | (Radespiel et al., 2001) |
| *Microcebus murinus* | Density | 130 | (Radespiel et al., 2001) |
| *Microcebus murinus* | Density | 146.96 | (Radespiel, 2000) |
| *Microcebus murinus* | Density | 169.82 | (Radespiel, 2000) |
| *Microcebus murinus* | Density | 220 | (Ausilio & Raveloanrinoro, 1998) |
| *Microcebus murinus* | Density | 288 | (Ausilio & Raveloanrinoro, 1998) |
| *Microcebus murinus* | Density | 300 | (Ganzhorn, 1992) |
| *Microcebus ravelobensis* | Density | 8 | (Rakotondravony & Radespiel, 2009) |
| *Microcebus ravelobensis* | Density | 27 | (Rakotondravony & Radespiel, 2009) |
| *Microcebus ravelobensis* | Density | 37 | (Rakotondravony & Radespiel, 2009) |
| *Microcebus ravelobensis* | Density | 38 | (Rakotondravony & Radespiel, 2009) |
| *Microcebus ravelobensis* | Density | 64 | (Rakotondravony & Radespiel, 2009) |
| *Microcebus ravelobensis* | Density | 94 | (Steffens & Lehman, 2016) |
| *Microcebus ravelobensis* | Density | 146 | (Rakotondravony & Radespiel, 2009) |
| *Microcebus ravelobensis* | Density | 239 | (Rakotondravony & Radespiel, 2009) |
| *Microcebus ravelobensis* | Density | 244 | (Rakotondravony & Radespiel, 2009) |
| *Microcebus ravelobensis* | Density | 258 | (Rakotondravony & Radespiel, 2009) |
| *Microcebus ravelobensis* | Density | 346 | (Rakotondravony & Radespiel, 2009) |
| *Microcebus ravelobensis* | Density | 402 | (Rakotondravony & Radespiel, 2009) |
| *Microcebus ravelobensis* | Density | 441 | (Rakotondravony & Radespiel, 2009) |
| *Microcebus ravelobensis* | Density | 511 | (Rakotondravony & Radespiel, 2009) |
| *Microcebus ravelobensis* | Density | 667 | (Rakotondravony & Radespiel, 2009) |
| *Microcebus ravelobensis* | Density | 730 | (Weidt et al., 2004) |
| *Microcebus ravelobensis* | Density | 833 | (Rakotondravony & Radespiel, 2009) |
| *Microcebus ravelobensis* | Density | 938 | (Rakotondravony & Radespiel, 2009) |
| *Microcebus tavaratra* | Density | 29 | (Salmona et al., 2014) |
| *Microcebus tavaratra* | Density | 67 | (Hawkins et al., 1990) |
| *Microcebus tavaratra* | Density | 80 | (Meyler et al., 2012) |
| *Microcebus tavaratra* | Density | 106 | (Salmona et al., 2014) |
| *Microcebus tavaratra* | Density | 109 | (Salmona et al., 2014) |
| *Microcebus tavaratra* | Density | 119 | (Salmona et al., 2014) |
| *Microcebus tavaratra* | Density | 183 | (Salmona et al., 2014) |
| *Microcebus tavaratra* | Density | 197 | (Salmona et al., 2014) |
| *Microcebus tavaratra* | Density | 208 | (Meyler et al., 2012) |
| *Microcebus tavaratra* | Density | 236 | (Salmona et al., 2014) |
| *Microcebus tavaratra* | Density | 239 | (Salmona et al., 2014) |
| *Microcebus tavaratra* | Density | 325 | (Salmona et al., 2014) |
| *Microcebus rufus* | Density | 6 | (Rasolofoson et al., 2007) |
| *Microcebus rufus* | Density | 10 | (Sterling & McFadden, 2000) |
| *Microcebus rufus* | Density | 12 | (Rasolofoson et al., 2007) |
| *Microcebus rufus* | Density | 12 | (Rasolofoson et al., 2007) |
| *Microcebus rufus* | Density | 12.5 | (Sterling & McFadden, 2000) |
| *Microcebus rufus* | Density | 20 | (Sterling & McFadden, 2000) |
| *Microcebus rufus* | Density | 21 | (Rasolofoson et al., 2007) |
| *Microcebus rufus* | Density | 21 | (Sterling & McFadden, 2000) |
| *Microcebus rufus* | Density | 30.6 | (Rasolofoson et al., 2007) |
| *Microcebus rufus* | Density | 30.6 | (Rasolofoson et al., 2007) |
| *Microcebus rufus* | Density | 32 | (Rasolofoson et al., 2007) |
| *Microcebus rufus* | Density | 39 | (Rasolofoson et al., 2007) |
| *Microcebus rufus* | Density | 39 | (Sterling & Rakotoarison, 1998) |
| *Microcebus rufus* | Density | 42 | (Ganzhorn, 1988) |
| *Microcebus rufus* | Density | 47.7 | (Hawkins et al., 2005) |
| *Microcebus rufus* | Density | 48 | (Rasolofoson et al., 2007) |
| *Microcebus rufus* | Density | 60 | (Rasolofoson et al., 2007) |
| *Microcebus rufus* | Density | 106 | (Rasolofoson et al., 2007) |
| *Microcebus rufus* | Density | 110 | (Ganzhorn, 1992) |
| *Microcebus rufus* | Density | 110 | (Ganzhorn, 1988) |
| *Microcebus rufus* | Density | 375 | (Ralison, 2006b) |
| *Mirza coquereli* | Density | 16 | (Schäffler & Kappeler, 2014b) |
| *Mirza coquereli* | Density | 22 | (Schäffler & Kappeler, 2014b) |
| *Mirza coquereli* | Density | 24 | (Ralison, 2008) |
| *Mirza coquereli* | Density | 28 | (Schäffler & Kappeler, 2014b) |
| *Mirza coquereli* | Density | 30 | (Hladik et al., 1980) |
| *Mirza coquereli* | Density | 30 | (Hladik et al., 1980) |
| *Mirza coquereli* | Density | 30 | (Reed, 1999) |
| *Mirza coquereli* | Density | 50 | (Petter et al., 1971) |
| *Mirza coquereli* | Density | 66 | (Ausilio & Raveloanrinoro, 1998) |
| *Mirza coquereli* | Density | 81 | (Ralison, 2008) |
| *Mirza coquereli* | Density | 96 | (Ralison, 2008) |
| *Mirza coquereli* | Density | 99 | (Ausilio & Raveloanrinoro, 1998) |
| *Mirza coquereli* | Density | 100 | (Bousquet & Rabetaliana, 1992) |
| *Mirza coquereli* | Density | 124 | (Ralison, 2008) |
| *Mirza coquereli* | Density | 126 | (Schäffler & Kappeler, 2014b) |
| *Mirza coquereli* | Density | 193 | (Ralison, 2008) |
| *Mirza coquereli* | Density | 206 | (Ralison, 2008) |
| *Mirza coquereli* | Density | 210 | (Petter et al., 1971) |
| *Phaner pallescens* | Density | 16 | (Ausilio & Raveloanrinoro, 1998) |
| *Phaner pallescens* | Density | 20 | (Schäffler & Kappeler, 2014b) |
| *Phaner pallescens* | Density | 30 | (Schäffler & Kappeler, 2014b) |
| *Phaner pallescens* | Density | 32 | (Schäffler & Kappeler, 2014b) |
| *Phaner pallescens* | Density | 37 | (Ralison, 2008) |
| *Phaner pallescens* | Density | 40 | (Hladik et al., 1980) |
| *Phaner pallescens* | Density | 44 | (Schäffler & Kappeler, 2014b) |
| *Phaner pallescens* | Density | 50 | (Hladik et al., 1980) |
| *Phaner pallescens* | Density | 50 | (Charles-Dominique & Petter, 1980) |
| *Phaner pallescens* | Density | 53 | (Ralison, 2008) |
| *Phaner pallescens* | Density | 55 | (Ralison, 2008) |
| *Phaner pallescens* | Density | 60 | (Ralison, 2008) |
| *Phaner pallescens* | Density | 100 | (Hladik et al., 1980) |
| *Phaner pallescens* | Density | 153 | (Ganzhorn & Kappeler, 1996) |
| *Phaner pallescens* | Density | 184 | (Ralison, 2008) |
| *Phaner pallescens* | Density | 205 | (Reed, 1999) |
| *Phaner pallescens* | Density | 206 | (Ralison, 2008) |
| *Phaner pallescens* | Density | 426 | (Ausilio & Raveloanrinoro, 1998) |
| *Phaner pallescens* | Density | 550 | (Petter et al., 1971) |
| *Phaner pallescens* | Density | 555 | (Ganzhorn & Kappeler, 1996) |
| *Phaner pallescens* | Density | 850 | (Petter et al., 1971) |
| *Propithecus diadema* | Density | 2.9 | (Hawkins et al., 2005) |
| *Propithecus diadema* | Density | 3.74 | (Rakotosamimanana et al., 2004) |
| *Propithecus diadema* | Density | 7.88 | (Rakotosamimanana et al., 2004) |
| *Propithecus diadema* | Density | 8 | (Ralison et al., 2015) |
| *Propithecus diadema* | Density | 10.75 | (Rakotosamimanana et al., 2004) |
| *Propithecus diadema* | Density | 11 | (Ralison, 2006b) |
| *Propithecus diadema* | Density | 14 | (Ralison, 2006b) |
| *Propithecus diadema* | Density | 24 | (Ralison et al., 2015) |
| *Propithecus diadema* | Density | 28 | (Ralison et al., 2015) |
| *Propithecus diadema* | Density | 40 | (Sterling & McFadden, 2000) |
| *Propithecus diadema* | Density | 90 | (Sterling & McFadden, 2000) |
| *Propithecus verreauxi* | Density | 1 | (Rasoarimanana, 2005) |
| *Propithecus verreauxi* | Density | 2 | (Rasoarimanana, 2005) |
| *Propithecus verreauxi* | Density | 2 | (Rasoarimanana, 2005) |
| *Propithecus verreauxi* | Density | 3 | (Rasoarimanana, 2005) |
| *Propithecus verreauxi* | Density | 5 | (Rasoarimanana, 2005) |
| *Propithecus verreauxi* | Density | 7 | (Rasoarimanana, 2005) |
| *Propithecus verreauxi* | Density | 8 | (Rasoarimanana, 2005) |
| *Propithecus verreauxi* | Density | 14 | (Rasoarimanana, 2005) |
| *Propithecus verreauxi* | Density | 19 | (Ganzhorn, 1992) |
| *Propithecus verreauxi* | Density | 20 | (Rasoarimanana, 2005) |
| *Propithecus verreauxi* | Density | 28 | (Rasoarimanana, 2005) |
| *Propithecus verreauxi* | Density | 29 | (Rasoarimanana, 2005) |
| *Propithecus verreauxi* | Density | 31 | (Rasoarimanana, 2005) |
| *Propithecus verreauxi* | Density | 34 | (Rasoarimanana, 2005) |
| *Propithecus verreauxi* | Density | 38 | (Rasoarimanana, 2005) |
| *Propithecus verreauxi* | Density | 39 | (Rasoarimanana, 2005) |
| *Propithecus verreauxi* | Density | 40 | (Rasoarimanana, 2005) |
| *Propithecus verreauxi* | Density | 46 | (Ganzhorn, 1992) |
| *Propithecus verreauxi* | Density | 53 | (Rasoarimanana, 2005) |
| *Propithecus verreauxi* | Density | 58 | (Rasoarimanana, 2005) |
| *Propithecus verreauxi* | Density | 61 | (Ausilio & Raveloanrinoro, 1998) |
| *Propithecus verreauxi* | Density | 76 | (Rasoarimanana, 2005) |
| *Propithecus verreauxi* | Density | 89.7 | (Jolly et al., 1982) |
| *Propithecus verreauxi* | Density | 98 | (Ausilio & Raveloanrinoro, 1998) |
| *Propithecus verreauxi* | Density | 104 | (Rasoarimanana, 2005) |
| *Propithecus verreauxi* | Density | 110 | (Rasoarimanana, 2005) |
| *Propithecus verreauxi* | Density | 115 | (Rasoarimanana, 2005) |
| *Propithecus verreauxi* | Density | 115 | (Rasoarimanana, 2005) |
| *Propithecus verreauxi* | Density | 115 | (Rasoarimanana, 2005) |
| *Propithecus verreauxi* | Density | 128 | (Rasoarimanana, 2005) |
| *Propithecus verreauxi* | Density | 150 | (Rasoarimanana, 2005) |
| *Propithecus verreauxi* | Density | 150 | (Rasoarimanana, 2005) |
| *Propithecus verreauxi* | Density | 230 | (Rasoarimanana, 2005) |
| *Propithecus verreauxi* | Density | 420 | (Rasoarimanana, 2005) |
| *Varecia variegata* | Density | 0.74 | (Rasolofoson et al., 2007) |
| *Varecia variegata* | Density | 0.90 | (Rasolofoson et al., 2007) |
| *Varecia variegata* | Density | 1 | (White et al., 1995) |
| *Varecia variegata* | Density | 1.11 | (Rakotosamimanana et al., 2004) |
| *Varecia variegata* | Density | 1.37 | (Rasolofoson et al., 2007) |
| *Varecia variegata* | Density | 1.45 | (Rakotosamimanana et al., 2004) |
| *Varecia variegata* | Density | 1.98 | (Rakotosamimanana et al., 2004) |
| *Varecia variegata* | Density | 3.70 | (Rasolofoson et al., 2007) |
| *Varecia variegata* | Density | 5 | (Ralison et al., 2015) |
| *Varecia variegata* | Density | 6.78 | (Rasolofoson et al., 2007) |
| *Varecia variegata* | Density | 8 | (Ralison et al., 2015) |
| *Varecia variegata* | Density | 8 | (Ralison et al., 2015) |
| *Varecia variegata* | Density | 8.53 | (Rasolofoson et al., 2007) |
| *Varecia variegata* | Density | 10.7 | (Rasolofoson et al., 2007) |
| *Varecia variegata* | Density | 11.1 | (Rasolofoson et al., 2007) |
| *Varecia variegata* | Density | 11.6 | (Rasolofoson et al., 2007) |
| *Varecia variegata* | Density | 16.2 | (White et al., 1995) |
| *Varecia variegata* | Density | 27 | (Ralison, 2006b) |
| *Varecia variegata* | Density | 34 | (Rasolofoson et al., 2007) |
| *Varecia variegata* | Density | 37 | (Ralison, 2006b) |
| *Varecia variegata* | Density | 175 | (Iwano, 1989) |

*Units of the parameters are: km (dispersal), year^-1^ (r) and ind/km^2^ (density).

Table S5. Empirical parameter values used in the empirical analyses, the average and 90%-confidence intervals of these parameter values (derived from allometric relationships) used in the allometric analyses, and the proportion of parameters for each species for which empirical values were found.

| Species | Parameter^a^ | Empirical value | Average value derived from allometric relationship | 90%-confidence interval of values derived from allometric relationships | Proportion of parameters with empirical values^b^ |
| --- | --- | --- | --- | --- | --- |
| *Avahi laniger* | Initial population density | 57.7 | 75.19 | 4.75 – 266.86 | 0.50 |
| *Avahi laniger* | Population density at carrying capacity | 112.7 | 196.64 | 14.18 – 726.72 | 0.50 |
| *Cheirogaleus major* | Initial population density | 44.5 | 66.21 | 5.96 – 247.04 | 0.50 |
| *Cheirogaleus major* | Population density at carrying capacity | 108.05 | 283.84 | 20.98 – 1236.13 | 0.50 |
| *Cheirogaleus medius* | r | 0.64 | 0.56 | 0.31 – 0.95 | 0.75 |
| *Cheirogaleus medius* | Initial population density | 139 | 113.37 | 8.22 – 440.37 | 0.75 |
| *Cheirogaleus medius* | Population density at carrying capacity | 483.6 | 434.04 | 22.49 – 1424.97 | 0.75 |
| *Eulemur albifrons* | Initial population density | 31.8 | 39.05 | 3.20 – 145.49 | 0.50 |
| *Eulemur albifrons* | Population density at carrying capacity | 142.3 | 142.09 | 13.92 – 562.41 | 0.50 |
| *Eulemur collaris* | Initial population density | 9 | 40.11 | 2.56 – 142.98 | 0.50 |
| *Eulemur collaris* | Population density at carrying capacity | 28.3 | 131.86 | 10.11 – 475.36 | 0.50 |
| *Eulemur macaco* | r | 0.3 | 0.31 | 0.17 – 0.53 | 0.25 |
| *Eulemur rubriventer* | Initial population density | 11.85 | 36.15 | 3.38 – 108.06 | 0.50 |
| *Eulemur rubriventer* | Population density at carrying capacity | 40.43 | 130.01 | 11.65 – 505.89 | 0.50 |
| *Eulemur rufus* | Initial population density | 46 | 40.95 | 3.09 – 126.96 | 0.50 |
| *Eulemur rufus* | Population density at carrying capacity | 178.8 | 125.81 | 14.53 – 377.95 | 0.50 |
| *Hapalemur griseus* | Initial population density | 6.9 | 56.14 | 4.59 – 191.85 | 0.50 |
| *Hapalemur griseus* | Population density at carrying capacity | 58.4 | 192.94 | 14.87 – 603.83 | 0.50 |
| *Indri indri* | Initial population density | 7.51 | 29.37 | 1.63 – 102.77 | 0.50 |
| *Indri indri* | Population density at carrying capacity | 17.275 | 111.96 | 4.72 – 417.24 | 0.50 |
| *Lemur catta* | r | 0.27 | 0.30 | 0.17 – 0.49 | 0.75 |
| *Lemur catta* | Initial population density | 106.125 | 34.43 | 2.70 – 115.56 |  |
| *Lemur catta* | Population density at carrying capacity | 265.9 | 133.77 | 11.02 – 455.75 |  |
| *Lepilemur mustelinus* | Initial population density | 38 | 51.30 | 3.38 – 176.84 | 0.50 |
| *Lepilemur mustelinus* | Population density at carrying capacity | 120 | 174.82 | 14.88 – 867.97 | 0.50 |
| *Microcebus berthae* | Initial population density | 119 | 273.10 | 18.05 – 847.31 | 0.50 |
| *Microcebus berthae* | Population density at carrying capacity | 724.8 | 724.55 | 63.01 – 2415.06 | 0.50 |
| *Microcebus danfossi* | Initial population density | 370 | 192.84 | 18.63 – 697.34 | 0.50 |
| *Microcebus danfossi* | Population density at carrying capacity | 490 | 622.00 | 57.82 – 2239.84 | 0.50 |
| *Microcebus griseorufus* | Initial population density | 320 | 203.32 | 15.77 – 693.85 | 0.50 |
| *Microcebus griseorufus* | Population density at carrying capacity | 1113.7 | 583.39 | 40.99 – 1658.70 | 0.50 |
| *Microcebus murinus* | r | 0.67 | 0.76 | 0.40 – 1.21 | 0.75 (no-dispersal scenario)  0.80 (dispersal scenario) |
| *Microcebus murinus* | Initial population density | 100 | 171.35 | 14.43 – 492.43 | 0.75 (no-dispersal scenario)  0.80 (dispersal scenario) |
| *Microcebus murinus* | Population density at carrying capacity | 292.8 | 619.54 | 65.50 – 1644.23 | 0.75 (no-dispersal scenario)  0.80 (dispersal scenario) |
| *Microcebus murinus* | Dispersal | 0.157 | 0.317 | 0.033 – 0.960 | 0.75 (no-dispersal scenario)  0.80 (dispersal scenario) |
| *Microcebus ravelobensis* | Initial population density | 251 | 231.78 | 17.50 – 658.98 | 0.50 |
| *Microcebus ravelobensis* | Population density at carrying capacity | 848.75 | 624.52 | 36.58 – 2008.85 | 0.50 |
| *Microcebus rufus* | Initial population density | 32 | 192.35 | 16.37 – 604.09 | 0.50 |
| *Microcebus rufus* | Population density at carrying capacity | 110 | 591.68 | 41.94 – 1702.60 | 0.50 |
| *Microcebus tavaratra* | Initial population density | 151 | 241.27 | 15.01 – 709.03 | 0.50 |
| *Microcebus tavaratra* | Population density at carrying capacity | 277.7 | 617.99 | 49.60 – 2068.16 | 0.50 |
| *Mirza coquereli* | r | 0.56 | 0.52 | 0.28 – 0.86 | 0.75 |
| *Mirza coquereli* | Initial population density | 73.5 | 91.20 | 7.72 – 345.44 | 0.75 |
| *Mirza coquereli* | Population density at carrying capacity | 206.6 | 402.64 | 17.02 – 1430.54 | 0.75 |
| *Phaner pallescens* | Initial population density | 55 | 93.28 | 5.74 – 342.79 | 0.50 |
| *Phaner pallescens* | Population density at carrying capacity | 555 | 289.93 | 22.13 – 1125.45 | 0.50 |
| *Propithecus diadema* | Initial population density | 11 | 27.57 | 2.11 – 75.51 | 0.50 |
| *Propithecus diadema* | Population density at carrying capacity | 65 | 73.33 | 6.79 – 228.40 | 0.50 |
| *Propithecus verreauxi* | r | 0.24 | 0.25 | 0.13 – 0.40 | 0.75 |
| *Propithecus verreauxi* | Initial population density | 39.5 | 39.96 | 2.98 – 124.59 | 0.75 |
| *Propithecus verreauxi* | Population density at carrying capacity | 170 | 149.42 | 10.53 – 611.00 | 0.75 |
| *Varecia variegata* | r | 0.46 | 0.27 | 0.15 – 0.46 | 0.75 |
| *Varecia variegata* | Initial population density | 8 | 27.54 | 2.38 – 91.85 | 0.75 |
| *Varecia variegata* | Population density at carrying capacity | 37 | 107.39 | 9.99 – 425.99 | 0.75 |

^a^Units of the parameters are: year^-1^ (r) and ind/km^2^ (initial population density, population density at carrying capacity), and km (dispersal).
^b^Assuming a total of four parameters for each species (intrinsic population growth rate, environmental stochasticity, initial population density, and population density at carrying capacity) in the no-dispersal scenario and five parameters in the dispersal scenario, which was only calculated for *Microcebus murinus* (the only species for which we have an empirical value of the median dispersal distance).


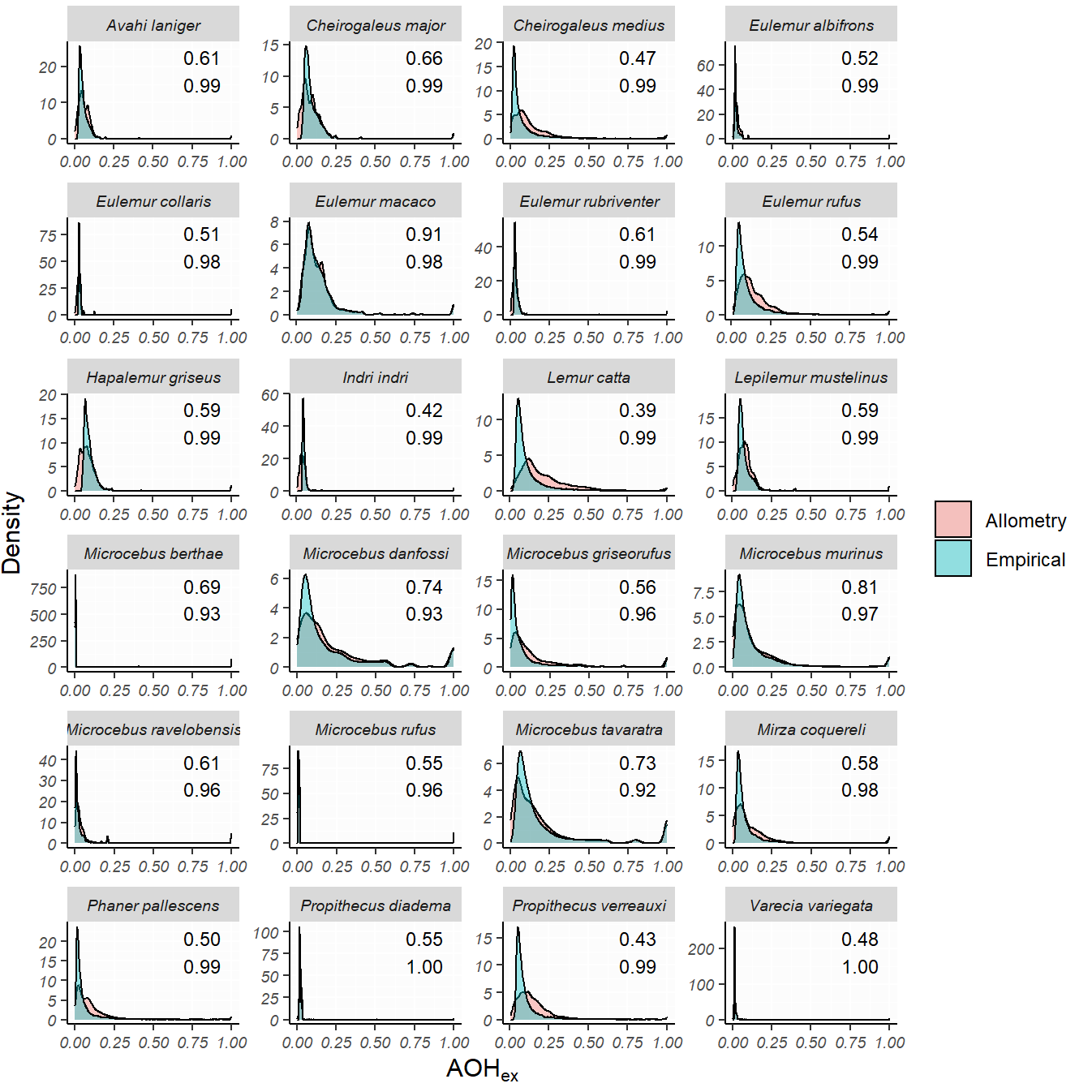


Fig. S7. Kernel density plots of the proportion of habitat area hosting populations committed to extirpation (AOH_ex_) in the no-dispersal scenario for the allometric (red) and empirical analyses (blue). Results are reported for 24 species for which empirical estimates were available for part of the population parameters. The first number in each panel indicate the proportional overlap between the kernel density distributions of the AOH_ex_ values from the allometric and empirical analyses. The second number indicates the proportion of AOH_ex_ values from the empirical analyses that fall within the range of values from the allometric analyses.


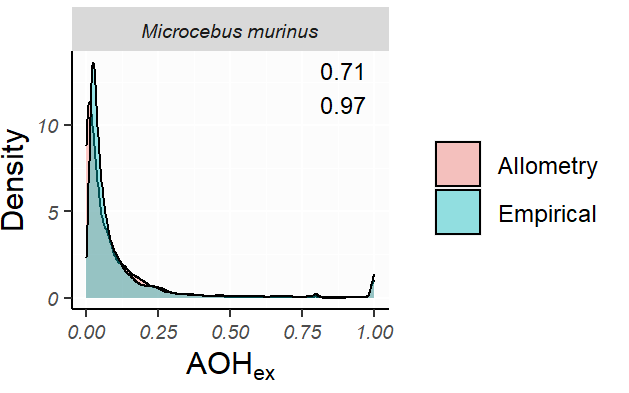


Fig. S8. Kernel density plot of the proportion of habitat area hosting populations committed to extirpation (AOH_ex_) in the dispersal scenario for the allometric (red) and empirical analyses (blue) for the grey mouse lemur (*Microcebus murinus*). The first number in the panel indicate the proportional overlap between the kernel density distributions of the AOH_ex_ values from the allometric and empirical analyses. The second number indicates the proportion of AOH_ex_ values from the empirical analyses that fall within the range of values from the allometric analyses.


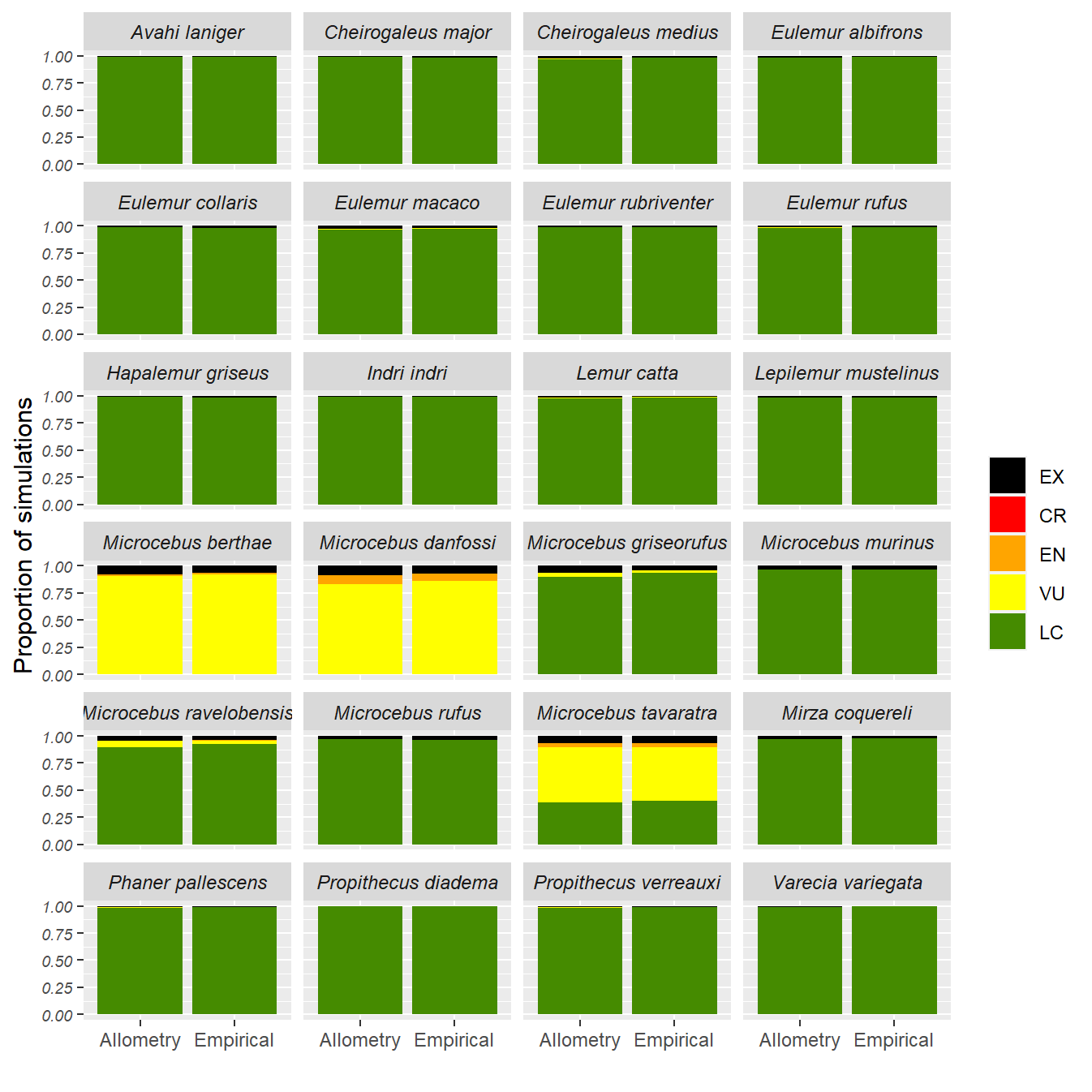


Fig. S9. Proportion of simulations in which a species is assigned a different International Union for the Conservation of Nature (IUCN) Red List category in the no-dispersal scenario for the allometric and empirical analyses. Results are reported for 24 species for which empirical estimates were available for part of the population parameters (LC, least concern; NT, near threatened; VU, vulnerable; EN, endangered; CR, critically endangered; EX, extinct).


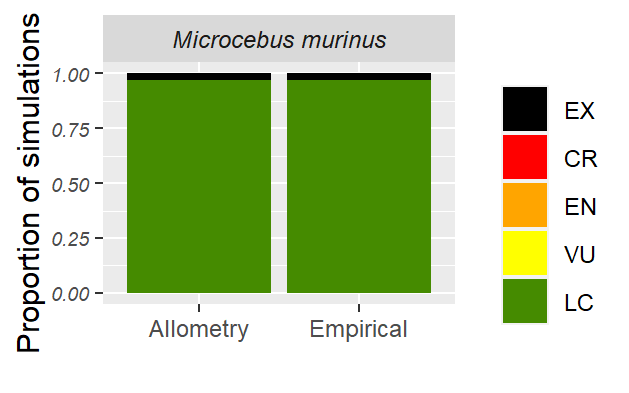


Fig. S10. Proportion of simulations in which the grey mouse lemur (*Microcebus murinus*) is assigned a different International Union for the Conservation of Nature (IUCN) Red List categories in the dispersal scenario for the allometric and empirical analyses (LC, least concern; NT, near threatened; VU, vulnerable; EN, endangered; CR, critically endangered; EX, extinct).

LITERATURE CITED

Ausilio E., Raveloanrinoro G. (1998) Les lémuriens de la région de Bemaraha: Forêts de Tsimembo, de l’Antsingy et de la région de Tsiandro. *Lemur News*, 3, 4-7.

Blanco M.B., Rasoazanabary E., Godfrey L.R. (2015) Unpredictable environments, opportunistic responses: Reproduction and population turnover in two wild mouse lemur species (Microcebus rufus and M. griseorufus) from eastern and western Madagascar. *American Journal of Primatology*, 77, 936-947.

Bohr Y.E.-M.B., Giertz P., Ratovonamana Y.R., Ganzhorn J.U. (2011) Gray-brown mouse lemurs (Microcebus griseorufus) as an example of distributional constraints through increasing desertification. *International Journal of Primatology*, 32, 901-913.

Bousquet B., Rabetaliana H. (1992) Site du patrimoine mondial des Tsingy de Bemaraha et autres sites d'intérêt biologique et écologique du Fivondronana d'Antsalova: évaluation et plan d'aménagement.

Charles-Dominique P., Petter J. (1980) Ecology and social life of Phaner furcifer. In 'Noctural Malagasy Primates'.(Eds P. Charles-Dominique et al.) pp. 75-95. Academic Press: New York.

Fietz J. (1999) Demography and floating males in a population of Cheirogaleus medius. Pages 159-172. *New directions in lemur studies*. Springer.

Ganzhorn J., Kappeler P. (1996) Lemurs of the Kirindy forest. *Primate report*, 46, 257-274.

Ganzhorn J.U. (1988) Food partitioning among Malagasy primates. *Oecologia*, 75, 436-450.

Ganzhorn J.U. (1992) Leaf chemistry and the biomass of folivorous primates in tropical forests. *Oecologia*, 91, 540-547.

Gardner C.J., Fanning E., Thomas H., Kidney D. (2009) The lemur diversity of the Fiherenana-Manombo Complex, southwest Madagascar. *Madagascar Conservation & Development*, 4.

Génin F. (2008) Life in unpredictable environments: first investigation of the natural history of Microcebus griseorufus. *International Journal of Primatology*, 29, 303-321.

Glessner K.D., Britt A. (2005) Population density and home range size of Indri indri in a protected low altitude rain forest. *International Journal of Primatology*, 26, 855-872.

Gould L., Sussman R.W., Sauther M.L. (1999) Natural disasters and primate populations: the effects of a 2-year drought on a naturally occurring population of ring-tailed lemurs (Lemur catta) in southwestern Madagascar. *International Journal of Primatology*, 20, 69-84.

Hawkins A., Chapman P., Ganzhorn J., Bloxam Q., Barlow S., Tonge S. (1990) Vertebrate conservation in Ankarana special reserve, northern Madagascar. *Biological Conservation*, 54, 83-110.

Hawkins A., Sterling E., Feistner A., Schmid J. (2005) Composition of the lemur community in the Vohibola III Classified Forest, SE Madagascar. *Lemur News*, 10, 16.

Hladik C.M., Charles-Dominique P., Petter J.-J. (1980) Feeding strategies of five nocturnal prosimians in the dry forest of the west coast of Madagascar. *Nocturnal Malagasy primates: ecology, physiology, and behavior*, 41-73.

Iwano T. (1989) Some observations of two kinds of Lemuridae (Varecia variegata variegata and Lemur fulvus albifrons) in the reserve of Nosy Mangabe. *Primates*, 30, 241-248.

Johnson S.E., Overdorff D.J. (1999) Census of brown lemurs (Eulemur fulvus sspp.) in southeastern Madagascar: Methods‐testing and conservation implications. *American Journal of Primatology: Official Journal of the American Society of Primatologists*, 47, 51-60.

Jolly A. (1985) Priorités dans l'étude des populations de Lémuriens. *Priorités en matière de conservation des espèces à Madagascar*, 59.

Jolly A. (1986) Lemur survival. Pages 71-98. *Primates: The Road to Self-Sustaining Populations*. Springer-Verlag, New York.

Jolly A., Dobson A., Rasamimanana H., Walker J., O'connor S., Solberg M., Perel V. (2002) Demography of Lemur catta at Berenty Reserve, Madagascar: effects of troop size, habitat and rainfall. *International Journal of Primatology*, 23, 327-353.

Jolly A., Gustafson H., Oliver W.L., O’Connor S.M. (1982) Propithecus verreauxi population and ranging at Berenty, Madagascar, 1975 and 1980. *Folia Primatologica*, 39, 124-144.

Meyler S.V., Salmona J., Ibouroi M.T., Besolo A., Rasolondraibe E., Radespiel U., Rabarivola C., Chikhi L. (2012) Density Estimates of Two Endangered Nocturnal Lemur Species From Northern M adagascar: New Results and a Comparison of Commonly Used Methods. *American Journal of Primatology*, 74, 414-422.

Müller P., Velo A., Raheliarisoa E.O., Zaramody A., Curtis D.J. (2000) Surveys of sympatric lemurs at Anjamena, north‐west Madagascar. *African Journal of Ecology*, 38, 248-257.

Murphy A.J., Farris Z.J., Karpanty S., Ratelolahy F., Kelly M.J. (2016) Estimating encounter rates and densities of three lemur species in northeastern Madagascar. *International Journal of Primatology*, 37, 371-389.

Overdorff D. (1988) Preliminary report on the activity cycle and diet of the red‐bellied lemur (Lemur rubriventer) in Madagascar. *American Journal of Primatology*, 16, 143-153.

Petter J., Schilling A., Pariente G. (1971) Observations Eco éthologiques sur deux lémuriens malgaches nocturnes: Phaner Furcifer et Microcebus Coquereli. *Revue d'Ecologie, Terre et Vie*, 287-327.

Radespiel L., Lutermann H., Schmelting B., Bruford M.W., Zimmermann E. (2003) Patterns and dynamics of sex-biased dispersal in a nocturnal primate, the grey mouse lemur, Microcebus murinus. *Animal Behaviour*, 65, 709-719.

Radespiel U. (2000) Sociality in the gray mouse lemur (Microcebus murinus) in northwestern Madagascar. *American Journal of Primatology: Official Journal of the American Society of Primatologists*, 51, 21-40.

Radespiel U., Ehresmann P., Zimmermann E. (2001) Contest versus scramble competition for mates: the composition and spatial structure of a population of gray mouse lemurs (Microcebus murinus) in north-west Madagascar. *Primates*, 42, 207-220.

Rakotondravony R., Radespiel U. (2009) Varying patterns of coexistence of two mouse lemur species (Microcebus ravelobensis and M. murinus) in a heterogeneous landscape. *American Journal of Primatology: Official Journal of the American Society of Primatologists*, 71, 928-938.

Rakotosamimanana B., Ralaiarison R., Ralisoamalala R., Rasolofoharivelo T.R., Raharimanantsoa V., Randrianarison R., Rakotondratsimba J., Rasolofoson D., Rakotonirainy E., Randriamboavonjy T. (2004) Comment et pourquoi les lémuriens diurnes disparaissent peu à peu dans les forêts d’Ambato et de Maromizaha (région de Moramanga) Madagascar. *Lemur News*, 9, 19-24.

Ralison J. (2006a) A lemur survey of the Réserve Spéciale de Marotandrano, Madagascar. *Lemur News*, 11, 35-37.

Ralison J. (2006b) Rapid assessment of lemurs in southern and southwestern forests of Madagascar. *Lemur News*, 11, 35-38.

Ralison J. (2008) Les lémuriens des forets sèches malgaches. *Les forêts sèches de Madagascar. Malagasy Nature*, 1, 135-156.

Ralison J., Rajaonson A., Ratsimbazafy J. (2015) Inventaire rapide des lémuriens de Maromizaha en vue d’un programme à long termes de suivi écologique participatif. *Lemur News*, 19, 21-24.

Ralison J.M. (2007) Lemur survey of the Andranomanitsy Forest, region of Besalampy, Province of Mahajanga. *Lemur News*, 12, 36-39.

Randrianambinina B., Rasoloharijaona S., Rakotondravony R., Zimmermann E., Radespiel U. (2010) Abundance and conservation status of two newly described lemur species in northwestern Madagascar (Microcebus danfossi, Lepilemur grewcockorum). *Madagascar Conservation & Development*, 5.

Rasoarimanana J. (2005) Suivi des Lémuriens diurnes dans le Parc National d’Andohahela. *Lemur News*, 10, 27-29.

Rasolofoson D., Rakotondratsimba G., Rakotonirainy O., Rasolofoharivelo T., Rakotozafy L., Ratsimbazafy J., Ratelolahy F., Andriamaholy V., Sarovy A. (2007) Le bloc forestier de Makira charnière de Lémuriens. *Lemur News*, 12, 49-53.

Reed K.E. (1999) Population density of primates in communities: differences in community structure. *Primate Communities, Cambridge University Press, Cambridge*, 116-140.

Ross C. (1988) The intrinsic rate of natural increase and reproductive effort in Primates. *Journal of Zoology*, 214, 199-219.

Ross C. (1992) Environmental correlates of the intrinsic rate of natural increase in primates. *Oecologia*, 90, 383-390.

Salmona J., Rakotonanahary A., Thani I., Zaranaina R., Ralantoharijaona T., Jan F., Rasolondraibe E., Barnavon M., Beck A., Wholhauser S. (2014) Estimation des densités et tailles de population du Microcèbe Roux du Nord de (Microcebus tavaratra) dans la région Loky-Manambato (Daraina). *Lemur News*, 18, 73-75.

Schäffler L., Kappeler P.M. (2014a) Distribution and abundance of the world's smallest primate, Microcebus berthae, in central western Madagascar. *International Journal of Primatology*, 35, 557-572.

Schäffler L., Kappeler P.M. (2014b) Distribution and abundance of three cheirogaleid species in Menabe Central, Western Madagascar. *Lemur News*, 18, 38-43.

Schwab D., Ganzhorn J.U. (2004) Distribution, population structure and habitat use of Microcebus berthae compared to those of other sympatric cheirogalids. *International Journal of Primatology*, 25, 307-330.

Steffens T.S., Lehman S.M. (2016) Factors determining Microcebus abundance in a fragmented landscape in Ankarafantsika National Park, Madagascar. *The dwarf and mouse lemurs of Madagascar: Biology, behavior and conservation biogeography of the Cheirogaleidae*, 73, 477-497.

Sterling E., McFadden K. (2000) Rapid census of lemur populations in the Parc National de Marojejy, Madagascar. *Fieldiana Zoology*, 265-274.

Sterling E., Rakotoarison N. (1998) Rapid assessment of richness and density of primate species on the Masoala peninsula, eastern Madagascar. *Folia Primatologica*, 69, 109-116.

Weidt A., Hagenah N., Randrianambinina B., Radespiel U., Zimmermann E. (2004) Social organization of the golden brown mouse lemur (Microcebus ravelobensis). *American Journal of Physical Anthropology*, 123, 40-51.

White F.J., Overdorff D.J., Balko E.A., Wright P.C. (1995) Distribution of ruffed lemurs (Varecia variegata) in Ranomafana National Park, Madagascar. *Folia Primatologica*, 64, 124-131.
